# CircRNA ARFGEF1 functions as a ceRNA to promote oncogenic KSHV-encoded viral interferon regulatory factor induction of cell invasion and angiogenesis by upregulating glutaredoxin 3

**DOI:** 10.1371/journal.ppat.1009294

**Published:** 2021-02-04

**Authors:** Shuihong Yao, Xuemei Jia, Fei Wang, Liuxue Sheng, Pengxia Song, Yanhui Cao, Hongjuan Shi, Weifei Fan, Xiangya Ding, Shou-Jiang Gao, Chun Lu

**Affiliations:** 1 Key Laboratory of Pathogen Biology of Jiangsu Province, Nanjing Medical University, Nanjing, P. R. China; 2 Department of Microbiology, Nanjing Medical University, Nanjing, P. R. China; 3 Medical School, Quzhou College of Technology, Quzhou, P. R. China; 4 State Key Laboratory of Reproductive Medicine, Department of Gynecology, Women’s Hospital of Nanjing Medical University, Nanjing Maternity and Child Health Hospital, Nanjing Medical University, Nanjing, P. R. China; 5 Department of Hematology and Oncology, Department of Geriatric Lung Cancer Research Laboratory, Geriatric Hospital of Nanjing Medical University, Nanjing, P. R. China; 6 UPMC Hillman Cancer Center, Department of Microbiology and Molecular Genetics, University of Pittsburgh, Pittsburgh, Pennsylvania, United States of America; Wistar Institute, UNITED STATES

## Abstract

Circular RNAs (circRNAs) are novel single-stranded noncoding RNAs that can decoy other RNAs to inhibit their functions. Kaposi’s sarcoma (KS), caused by oncogenic Kaposi’s sarcoma-associated herpesvirus (KSHV), is a highly angiogenic and invasive vascular tumor of endothelial origin commonly found in AIDS patients. We have recently shown that KSHV-encoded viral interferon regulatory factor 1 (vIRF1) induces cell invasion, angiogenesis and cellular transformation; however, the role of circRNAs is largely unknown in the context of KSHV vIRF1. Herein, transcriptome analysis identified 22 differentially expressed cellular circRNAs regulated by vIRF1 in an endothelial cell line. Among them, circARFGEF1 was the highest upregulated circRNA. Mechanistically, vIRF1 induced circARFGEF1 transcription by binding to transcription factor lymphoid enhancer binding factor 1 (Lef1). Importantly, upregulation of circARFGEF1 was required for vIRF1-induced cell motility, proliferation and *in vivo* angiogenesis. circARFGEF1 functioned as a competing endogenous RNAs (ceRNAs) by binding to and inducing degradation of miR-125a-3p. Mass spectrometry analysis demonstrated that glutaredoxin 3 (GLRX3) was a direct target of miR-125a-3p. Knockdown of GLRX3 impaired cell motility, proliferation and angiogenesis induced by vIRF1. Taken together, vIRF1 transcriptionally activates circARFGEF1, potentially by binding to Lef1, to promote cell oncogenic phenotypes via inhibiting miR-125a-3p and inducing GLRX3. These findings define a novel mechanism responsible for vIRF1-induced oncogenesis and establish the scientific basis for targeting these molecules for treating KSHV-associated cancers.

## Author summary

Kaposi’s sarcoma-associated herpesvirus (KSHV) is the etiological agent of Kaposi’s sarcoma (KS), which frequently occurs in people with AIDS. We and others had proved that KSHV-encoded viral interferon regulatory factor 1 (vIRF1) was crucial in the pathogenesis of KSHV-induced cancers. KSHV genome transcribes viral circular RNAs (circRNAs), however, the role of cellular circRNAs in vIRF1-induced tumorigenesis remains unknown. CircRNAs serves as competitive endogenous RNAs (ceRNAs) of miRNAs, thus regulating miRNA-mRNA network to influence mRNA stability and protein expression. Here we found that vIRF1 binds to the promoter of the parental gene ARFGEF1 and activate circARFGEF1 transcription through interaction with transcription factor lymphoid enhancer binding factor 1 (Lef1). CircARFGEF1 functioned as a ceRNA by binding to and inducing degradation of miR-125a-3p, thereby abrogating the inhibition effect of this miRNA on its direct targeting of GLRX3. Significantly, circARFGEF1/miR-125a-3p/GLRX3 axis was required for vIRF1 induction of cell motility, proliferation and *in vivo* angiogenesis. In summary, our study describes a novel mechanism of KSHV-induced oncogenesis by hijacking host circRNAs through a viral oncogene.

## Introduction

Kaposi’s sarcoma (KS), which arises in skin lesions, lymph nodes, mucous membranes and other organs, is the most common malignant tumor in patients with HIV/AIDS [1,2]. Skin KS is characterized by the abnormal angio-proliferative and metastasis of endothelium cells [2]. Kaposi’s sarcoma-associated herpesvirus (KSHV), also known as human herpesvirus 8 (HHV-8), is the etiological agent of KS [3]. Besides KS, KSHV also causes two B-cell lymphoproliferative disorders, primary effusion lymphoma (PEL) and multicentric Castleman’s disease (MCD), and KSHV-associated inflammatory cytokine syndrome (KICS) [4].

Like other herpesviruses, KSHV contains two life cycles, lytic replication and latency [5]. KSHV encodes several oncoproteins, which are essential for the development and progression of KS [6]. Among them, viral interferon regulatory factor 1 (vIRF1) is encoded by open reading frame (ORF)-K9, which shares ~25% homology to human cellular IRFs [7]. vIRF1 is a lytic gene [8]. We and others detected the expression of vIRF1 protein and mRNA in KSHV-infected cells in KS tumors [9,10]. Regarding immune response, vIRF1 competitively binds to IRFs to inhibit their transcriptional activity and attenuates the expression of IFN-induced genes [11,12]. Nevertheless, vIRF1 is not limited to the anti-viral immune response. vIRF1 suppresses p53 function and TGF-β/Smad signaling, participating in cancer regulatory network [13,14]. In addition, vIRF1 plays a key role in KSHV lytic replication. vIRF1 directly binds to a mitophagy receptor, NIX, on the mitochondria and activates NIX-mediated mitophagy to promote mitochondrial clearance, resulting in enhanced KSHV productive replication [15]. Although we and others have shown that vIRF1 promotes cell migration, invasion, proliferation, angiogenesis, cellular transformation and tumorigenesis *in vitro* and *in vivo* [9,12,16,17], the underlying mechanisms of vIRF1-mediated tumor pathogenesis still remain to be explored.

Circular RNA (circRNA), a new class of non-coding RNAs, is a kind of covalently closed-loop RNA without 3’ and 5’ ends [18]. circRNAs are the products of mRNA alternating spicing, occurring in homo sapiens, mouse, *C*. *elegans*, Drosophila, yeast and plants [19–21]. Recently, human cellular circRNAs were reported to play an crucial role in the occurrence and development of human diseases, especially in human cancers [22]. By RNA sequencing, KSHV was found to encode numerous back-spliced circRNAs including circvIRF4, circK7.3s and circPANs [23–26]. These three circRNAs could be packed into KSHV particles. Similar to Orf-K7.3 and PAN, circK7.3s and circPANs were induced upon KSHV lytic replication, and only circvIRF4 was constitutively expressed in KSHV infected cells [25,26]. Although cellular circRNAs regulated by KSHV had been identified in primary human umbilical vein endothelial cells (pri-HUVECs) and B cell line [24], whether cellular circRNAs were involved in KSHV vIRF1-induced tumorigenesis remains largely unknown.

To date, most studies demonstrate that cellular circRNAs function as competing endogenous RNAs (ceRNAs) to sponge miRNAs, resulting in the elevation of miRNAs targeted genes [19,27]. For example, circular RNA cESRP1 inhibited TGF-β pathway by sponging miR-93-5p to sensitize cells of small cell lung cancer to chemotherapy [28]. However, circRNAs are not limited to act as sponges for miRNAs. circRNA-protein interaction exhibits another possibility to regulate cellular process. For instance, circAGO2, a circRNA generated from AGO2 gene, directly interacts with HuR, which activates HuR to mediate AGO2/miRNA dependent gene silencing to drive cancer progression [28].

In this study, we aimed to unfold the role of vIRF1-regulated cellular circRNAs in the tumorigenesis of KS and the underlying mechanisms. We identified vIRF1-regulated circRNAs through RNA sequencing in endothelial cells infected by KSHV and a vIRF1 mutant virus. Six circRNAs increased by vIRF1 and 16 circRNAs decreased by vIRF1 were authenticated and analyzed by bioinformatics tools. Among them, hsa_circ_0001808, which was named circARFGEF1 in this study, is generated from the ARFGEF1 gene locus. vIRF1-activated circARFGEF1 transcription by interacting with transcription factor lymphoid enhancer binding factor 1 (Lef1). Furthermore, the decreased miR-125a-3p was sponged by circARFGEF1. Mass spectrometry analysis and luciferase reporter assay demonstrated that GLRX3 was a direct target of miR-125a-3p. More importantly, circARFGEF1/miR-125a-3p/GLRX3 axis was involved in vIRF1-induced cell motility, proliferation and *in vivo* angiogenesis. In conclusion, our results imply that vIRF1-modulated circARFGEF1/miR-125a-3p/GLRX3 axis is crucial for the pathogenesis of KSHV-induced tumors, which could be a potential molecular therapeutic target for treating KSHV-related diseases.

## Results

### Identification of KSHV vIRF1-regulated circRNAs by RNA-sequencing

**Fig 1:**
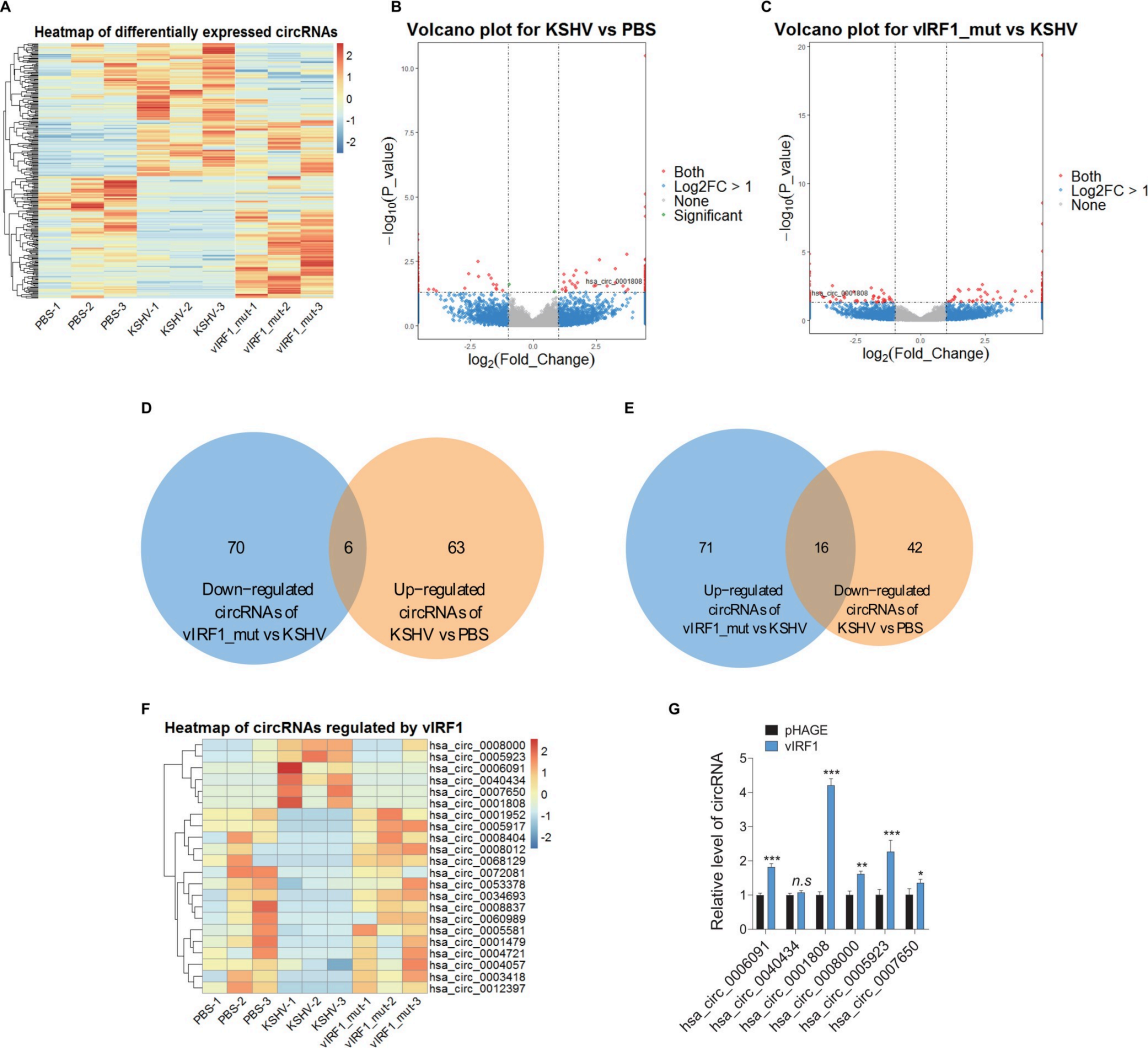
circRNA expression profiles in vIRF1 transduced and KSHV infected endothelial cell line. **(A)**. Heatmap analyses of RNA-seq showing differentially expressed circRNAs. PBS treated, KSHV_WT virus (3 MOI) infected or vIRF1_mut virus infected endothelial cell line EA.hy926 for 72 h were collected and subjected to RNA sequencing and pseudo-color represented the intensity scale of dysregulated circRNAs. **(B)**. Volcano plots illustrate the differentially expressed circRNAs in KSHV group compared to PBS group. DESeq method was used to adjust p-values; the p-value fold cut off for significance is less than 0.05. **(C)**. Volcano plots illustrate the differentially expressed circRNAs in vIRF1_mut group compared to KSHV group. DESeq method was used to adjust p-values; the p-value fold cut off for significance is less than 0.05. **(D)**. Venn diagram analysis shows 6 vIRF1 up-regulated circRNAs after comparing KSHV and vIRF1_mut dysregulated circRNAs. **(E)**. Venn diagram analysis shows 16 vIRF1 down-regulated circRNAs after comparing KSHV and vIRF1_mut dysregulated circRNAs. **(F)**. Clustering and detailed analysis of 22 vIRF1-regulated circRNAs for the reliability and stability of differential expression and sample quality. Pseudo-color represented the intensity scale of dysregulated circRNAs. **(G)**. RT-qPCR validation of 6 vIRF1 up-regulated circRNAs from RNA-seq in endothelial cell line EA.hy926 transduced with 2 MOI of lentiviral vIRF1 or its control pHAGE. Data were shown as mean ± SD. * *P* < 0.05, ** *P* < 0.01, and *** *P* < 0.001, statistical significance was determined using Student’s t-test. *n*.*s*, not significant.

To identify cellular circRNAs regulated by KSHV vIRF1, an endothelial cell line EA.hy926 was either treated with PBS, or infected with 3 MOI of KSHV_WT virus or vIRF1_mut virus for 3 days [16,17]. We characterized circRNAs profiles in these cells by total RNA-sequencing. Cluster analysis showed that cells infected by KSHV_WT had a circRNA expression pattern distinct from those infected by the vIRF1_mut virus (Fig 1A). The Volcano Plots revealed the differential distributions of circRNAs in KSHV_WT and vIRF1_mut virus-infected cells compared to those of PBS and KSHV groups, respectively (Fig 1B and 1C). There were 127 differentially expressed circRNAs in KSHV_WT virus-infected cells compared to the PBS group, and 163 differentially expressed circRNAs in KSHV_WT virus-infected cells compared to the vIRF1_mut virus-infected cells (Fig 1D and 1E). We identified 22 circRNAs including 6 vIRF1-upregulated and 16 vIRF1-downregulated circRNAs (Fig 1D and 1E). Clustering analysis showed that differential expression and sample quality of these 22 vIRF1-regulated circRNAs were reliable and stable (Fig 1F).

To examine the expression of vIRF1-regulated circRNAs, we first established ectopic vIRF1-expressing EA.hy926 cells by transduction with 2 MOI of lentiviral vIRF1. We found that at this MOI vIRF1-transduced cells showed a vIRF1 mRNA level similar to that of KSHV-infected cells (S1 Fig). The lentiviral vIRF1 was generated in our recent study [16]. RT-qPCR was performed to validate 6 vIRF1-upregulated circRNAs in RNA-sequencing. We found that hsa_circ_0001808 was increased at the highest level in vIRF1-transduced cells compared to control lentiviral pHAGE (Fig 1G). According to the human reference genome, hsa_circ_0001808, which was located at chr8: 2966846–2983691, and derived from gene ARFGEF1, was termed as “circARFGEF1”.

### vIRF1-Lef1 complex activates circARFGEF1 transcription to promote cell motility and angiogenesis

**Fig 2:**
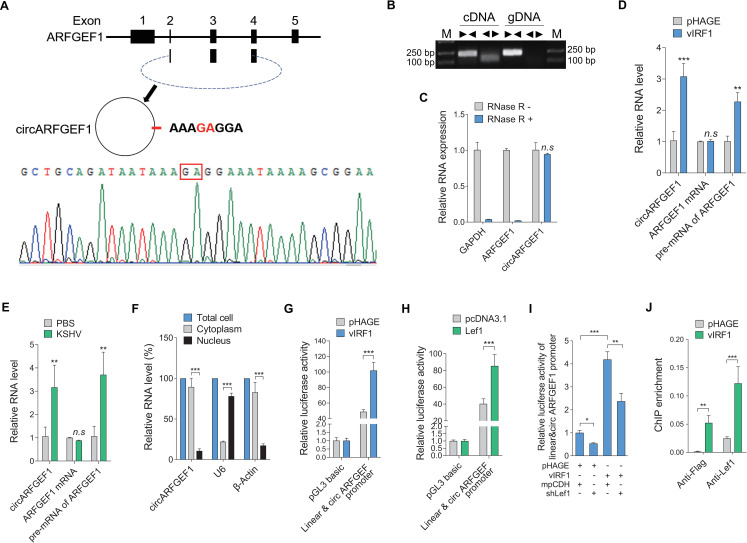
vIRF1/Lef1 complex activates circARFGEF1 transcription. **(A).** circARFGEF1 was generated from the parental ARFGEF1 gene and consisted of the head-to-tail splicing of exon 2, 3 and 4 (335 nt) (upper and middle panels). Sanger sequencing results showed the back-splice junction sequences of circARFGEF1 (lower panel). **(B)**. circARFGEF1 sequences that were PCR-amplified from cDNA and gDNA with divergent and convergent primers. **(C)**. RNAse R treatment assay was performed, and only circARFGEF1 showed exonuclease resi but not the linear ARFGEF1 mRNA. **(D).** RT-qPCR was performed to detect circARFGEF1, ARFGEF1 mRNA and pre-mRNA levels of ARFGEF1 gene in vIRF1 transduced endothelial cell line EA.hy926. circARFGEF1 and pre-mRNA of ARFGEF1 were significantly increased in vIRF1 transduced cells compared to the control group. But there were no changes on ARFGEF1 mRNA in vIRF1 transduced cells. **(E).** RT-qPCR was performed to examine circARFGEF1, ARFGEF1 mRNA and pre-mRNA levels of ARFGEF1 gene in KSHV (3 MOI) infected endothelial cell line EA.hy926. circARFGEF1 and pre-mRNA of ARFGEF1 were significantly increased in KSHV infected cells compared to the control group. But there were no changes on ARFGEF1 mRNA in KSHV infected cells. **(F)**. circARFGEF1 is the most enriched in cytoplasm. Levels of circARFGEF1, U6 and β-actin in purified nuclear and cytoplasm fractions of endothelial cell line were examined by RT-qPCR. **(G)**. Luciferase reporter activity in HEK293T cells co-transfected with vIRF1 and circARFGEF1 promoter constructs for 48 h. vIRF1 significantly increased circARFGEF1 promoter activity compared to that of control lentiviral pHAGE. **(H)**. Luciferase reporter activity in HEK293T cells co-transfected with Lef1 and circARFGEF1 promoter constructs for 48 h. Lef1 significantly increased circARFGEF1 promoter activity compared to that of control pcDNA3.1. **(I)**. Luciferase reporter activity in vIRF1-transduced EA.hy926 cells co-transfected with shLef1 and circARFGEF1 promoter constructs for 48 h. **(J).** The binding of vIRF1 and Lef1 to circARFGEF1 promoter was examined by ChIP-qPCR. vIRF1-transduced EA.hy926 cells were collected and subjected to ChIP-qPCR with anti-Flag (vIRF1) and anti-Lef1 antibodies, respectively. Data were shown as mean ± SD. * *P* < 0.05, ** *P* < 0.01, and *** *P* < 0.001; *n*.*s*, not significant. Statistical significance was determined using Student’s t-test in C, D, E, F and J; one-way ANOVA followed by Tukey’s multiple comparisons test in G, H and I.

As annotated in circBase (www.circbase.org), circARFGEF1 arises from the ARFGEF1 gene and consists of the head-to-tail splicing of exon 2, 3 and 4 (335 nt) (Fig 2A, upper and middle panels). The back-splice junctions of circARFGEF1 were confirmed by Sanger sequencing (Fig 2A, lower panel). To verify the endogenous circARFGEF1 generated from the exons of ARFGEF1 gene, we designed divergent and convergent primers that specifically amplified the back-spliced and canonical form of ARFGEF1. With divergent primers, circARFGEF1 was amplified by RT-PCR from cDNA template but not gDNA (Fig 2B). Further, circARFGEF1 rather than linear *ARFGEF1* was resistant to RNase R digestion, which degrades linear RNAs but does not act on circular RNA (Fig 2C). Besides being increased in vIRF1-transduced cells (Figs 1G and 2D), circARFGEF1 was also induced in KSHV-infected cells (Fig 2E). To compare the expression between circARFGEF1 and linear *ARFGEF1* or pre-mRNA of ARFGEF1, we generated specific primers that targeted the different RNA molecules. The results showed that pre-mRNA of ARFGEF1 was increased in both vIRF1-transduced and KSHV-infected cells, whereas KSHV or vIRF1 has no effect on linear *ARFGEF1* (Figs 2D and 2E and S2). Consistent with these observations, neither vIRF1 transduction nor KSHV infection affected the protein level of ARFGEF1 in EA.hy926 cells (S3A and S3B Fig).

Like most circRNAs, circARFGEF1 is mostly localized to the cytoplasm (Fig 2F). To examine whether vIRF1 regulates the transcription of circARFGEF1, we performed a dual-luciferase reporter gene assay. We found that vIRF1 activated circARFGEF1 promoter activity (Fig 2G). Our recent studies reported that vIRF1 interacted with transcription factor Lef1 to bind to the promoters of CDCP1 and SPAG9 genes, and enhance their transcriptions and expressions [9,17]. Here, we first overexpressed Lef1, and found that circARFGEF1 transcription was significantly enhanced by Lef1 (Fig 2H). Knockdown of the Lef1 with a mixture of lentivirus-mediated short hairpin RNAs (shRNAs) targeting Lef1 dramatically impaired the activation of effect of vIRF1 on the circARFGEF1 promoter in a luciferase reporter assay (Fig 2I). To confirm the physical interaction of Lef1 with the sequence of circARFGEF1 promoter, the chromatin immunoprecipitation assay (ChIP) was performed. We observed that binding of Lef1 to circARFGEF1 promoter was increased in the presence of vIRF1 (Fig 2J).

**Fig 3:**
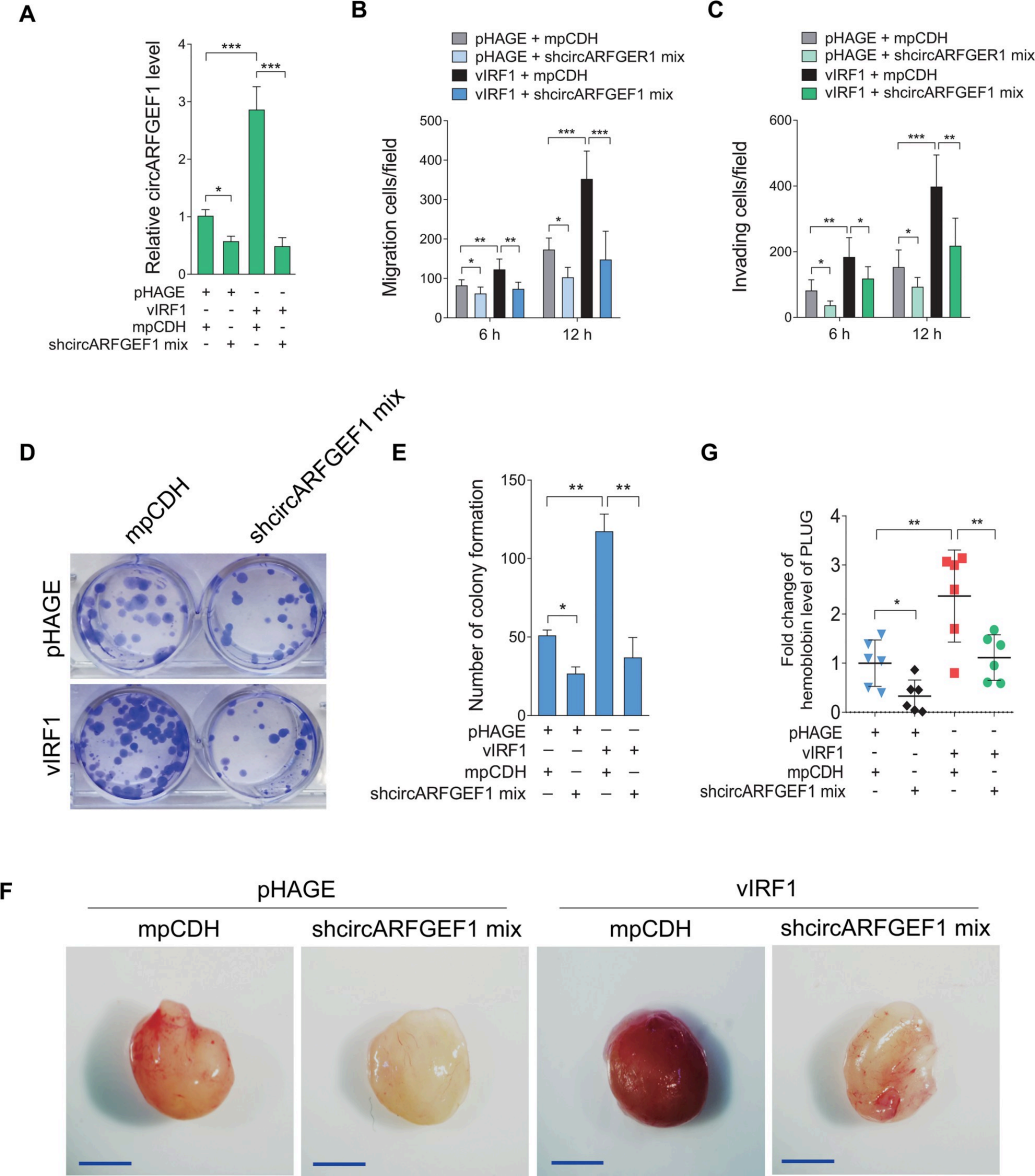
circARFGEF1 promotes vIRF1-induced cell motility, proliferation and angiogenesis. **(A)**. RT-qPCR analysis of circARFGEF1 expression in vIRF1-expressing EA.hy926 cells transduced with a mixture of shRNAs targeting circARFGEF1 (shcircARFGEF1 mix). **(B)**. Migration analysis of pri-HUVECs treated as in (A). Transwell migration assay was performed as described in the “Materials and methods” section. **(C)**. Invasion analysis of pri-HUVECs treated as in (A). Transwell invasion assay was performed as described in the “Materials and methods” section. **(D)**. Plate colony formation assay of endothelial cell line EA.hy926 treated as in (A). Plate colony formation assay was performed as described in the “Materials and methods” section. **(E)**. Quantification of cell colony formation described in (D). **(F)**. The mixture containing high concentration Matrigel and endothelial cell line EA.hy926 treated as in (A) was injected into nude mice. Representational photographs of plugs were exhibited. Scar bars, 1 cm. **(G)**. The level of hemoglobin in plug tissues treated in (F) was measured by comparing the standard curve. Data were shown as mean ± SD. * *P* < 0.05, ** *P* < 0.01, and *** *P* < 0.001, *n*.*s*, not significant. Statistical significance was determined using one-way ANOVA followed by Tukey’s multiple comparisons test.

Our recent reports showed that vIRF1 promoted endothelial cell migration, invasion, proliferation, angiogenesis and cellular transformation [9,16,17]. We asked whether vIRF1-upregulated circARFGEF1 was involved in this process. To exclude the effect of the parental gene ARFGEF1 of circARFGEF1 on these cell malignant phenotypes, we examined ARFGEF1 expression after lentivirus-mediated overexpression or knockdown of circARFGEF1. We found that either overexpression or silencing of circARFGEF1 didn’t affect the mRNA level of its parental gene ARFGEF1 (S4 and S5 Figs). Loss of circARFGEF1 significantly inhibited vIRF1-induced endothelial cell migration and invasion (Figs 3A–3C and S6). Similarly, knockdown of circARFGEF1 impeded vIRF1-induced cell proliferation in a plate colony formation assay (Fig 3D and 3E), while the inhibitory effect on the plate colony formation by silencing of circARFGEF1 was reversed by overexpression of circARFGEF1 in EA.hy926 cells (S7 Fig). To examine the effect of circARFGEF1 on *in vivo* angiogenesis, matrigel plug assay in mice was performed. Knockdown of circARFGEF1 impaired vIRF1-induced angiogenesis (Fig 3F and 3G). Taken together, these data suggest that vIRF1-Lef1 complex enhances circARFGEF1 transcription to promote vIRF1-induced cell motility, proliferation and angiogenesis.

### circARFGEF1 binds to and degrades miR-125a-3p to promote vIRF1-induced cell invasion and angiogenesis

**Fig 4:**
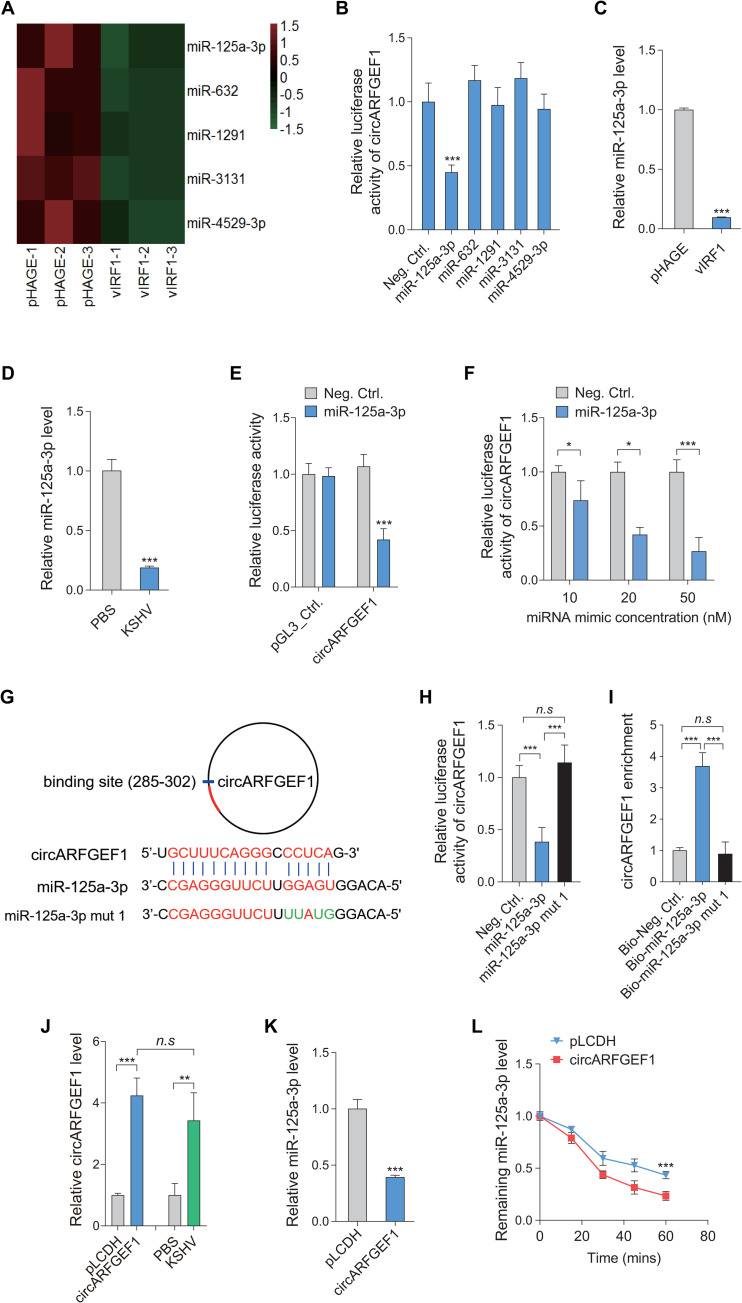
circARFGEF1 targets miR-125a-3p and reduces its stability. **(A)**. Heatmap analyses of miRNA array showing downregulated miRNAs with circARFGEF1 binding potential in vIRF1 transduced cells. Lentivirus pHAGE transduced cells were used as a control and pseudo-color represented the intensity scale of downregulated miRNAs. **(B)**. CircARFGEF1 sequence was cloned into the downstream of luciferase gene in pGL3-control plasmid to generate pGL3-circARFGEF1 luciferase reporter plasmid. Mimics of five candidate miRNAs (20 nM) as predicted by data in panel A or miRNA negative control (Neg. Ctrl.) were co-transfected with pGL3-circARFGEF1 luciferase reporter into HEK293T cells for luciferase reporter assays for 48 h. **(C)**. RT-qPCR was employed to examine miR-125a-3p level in vIRF1 transduced EA.hy926 cells. Lentivirus pHAGE transduced cells were used as a control. **(D)**. RT-qPCR was used to detect miR-125a-3p level in KSHV (3 MOI) infected EA.hy926 cells. PBS-treated cells were used as a control. **(E)**. Effects of miR-125a-3p on the luciferase reporter activity of the pGL3-circARFGEF1 and the control reporter pGL3_Control. HEK293T cells were transfected with miR-125a-3p (miR-125a-3p) mimics (20 nM) or miRNA negative control (Neg. Ctrl.) along with pGL3_Control or pGL3-circARFGEF1 reporter plasmid for 48 h. **(F)**. Luciferase assay of the pGL3-circARFGEF1 reporter co-transfected with increasing amounts of miRNA negative control (Neg. Ctrl.) or a mimic of miR-125a-3p (10, 20, and 50 nM) in HEK293T cells for 48 h. **(G)**. Putative binding site of miR-125a-3p in the circARFGEF1 sequence and mutagenesis of target site in miR-125a-3p (miR-125a-3p mut 1) were shown. **(H)**. Luciferase assay of the pGL3-circARFGEF1 reporter co-transfected with miR-125a-3p mimic (miR-125a-3p), miR-125a-3p mutant mimic (miR-125a-3p mut 1) or miRNA negative control (Neg. Ctrl.) in HEK293T cells for 48 h. **(I)**. RNA pull-down analysis was performed with biotin-labeled miRNA and its control including bio-Neg. Ctrl., bio-miR-125a-3p and bio-miR-125a-3p mut 1 in EA.hy926 cells. Specific primers were used to detect the enrichment of circARFGEF1. **(J)**. RT-qPCR analysis of circARFGEF1 expression efficiency and the level comparison to KSHV infected cells. EA.hy926 cells were transduced with lentivirus circARFGEF1 (1 MOI) and its control pLCDH, or infected with KSHV (3 MOI) or treated with PBS, and were collected for RT-qPCR for detection of circARFGEF1 level. **(K)**. RT-qPCR analysis of miR-125a-3p expression. EA.hy926 cells were transduced with lentivirus circARFGEF1 and its control pLCDH, and were collected for RT-qPCR for detection of miR-125a-3p level. **(L)**. RT-qPCR analysis of the level of miR-125a-3p with overexpression of circARFGEF1. EA.hy926 cells transduced with lentivirus circARFGEF1 and its control pLCDH were treated with Actinomycin D for 20, 40 60 min, and were collected for RT-qPCR for detection of miR-125a-3p level. Data were shown as mean ± SD. * *P* < 0.05, ** *P* < 0.01, and *** *P* < 0.001; *n*.*s*, not significant. Statistical significance was determined using Student’s t-test in B, C, D, F and K; one-way ANOVA followed by Tukey’s multiple comparisons test in E, H, I and J; two-way ANOVA in L.

CircRNAs are known to function as competitive endogenous RNAs (ceRNAs) to sponge miRNAs [29]. To identify the miRNA(s) that involved in the vIRF1-circARFGEF1 network, we analyzed our previous miRNA data generated from vIRF1 transduced cells (GEO accession number: GSE119034) [16]. The Basic Local Alignment Search Tool (BLAST) was used to predict potential binding between circARFGEF1 and vIRF1-downregulated miRNAs. As a result, there were five miRNAs, which exhibited the potential binding to circARFGEF1 (Fig 4A). To validate the interaction between miRNAs and circARFGEF1, we constructed a reporter plasmid, where the circARFGEF1 sequence was cloned into the downstream of luciferase gene in the pGL3-control plasmid, and co-transfected the reporter plasmid with each of the five miRNA mimics into 293T cells, respectively. A dual luciferase reporter system was performed to validate the binding of miRNA and circARFGEF1. It was showed that only miR-125a-3p reduced circARFGEF1 luciferase activity (Fig 4B). RT-qPCR indicated that miR-125a-3p was decreased in both vIRF1-transduced and KSHV-infected EA.hy926 cells (Fig 4C and 4D). The extents of miR-125a-3p reduction detected by microarray and qPCR methods varied, which could be due to the nature of the two different methods; however, they were in the same scale. miR-125a-3p inhibited circARFGEF1 luciferase activity in a dose-dependent manner in a luciferase reporter assay (Fig 4E and 4F). To examine the binding site located in circARFGEF1 sequence, we generated the mutated miR-125a-3p according to the sequences of putative binding site (Fig 4G). miR-125a-3p mutation on the seed sequence failed to inhibit the circARFGEF1 reporter activity (Fig 4H). To determine whether the interaction between circARFGEF1 and miR-125a-3p existed in cells, we generated biotin-labeled wild type (WT) of miR-125a-3p and mutated miR-125a-3p. Biotin-coupled RNA pull-down assay showed that circARFGEF1 was enriched in miR-125a-3p WT group but not in mutated group (Fig 4I). We further examined the expression level of miR-125a-3p following overexpression of circARFGEF1 (1 MOI) at a level comparable that of KSHV-infected EA.hy926 cells (Figs 4J and S4A). We found that the level of miR-125a-3p was dramatically decreased after overexpression of circARFGEF1 (Fig 4K). More importantly, miR-125a-3p was degraded in circARFGEF1-expressing cells in response to actinomycin D (Fig 4L). Collectively, these data suggest that circARFGEF1 served as a sponge of miR-125a-3p, inducing its degradation.

**Fig 5:**
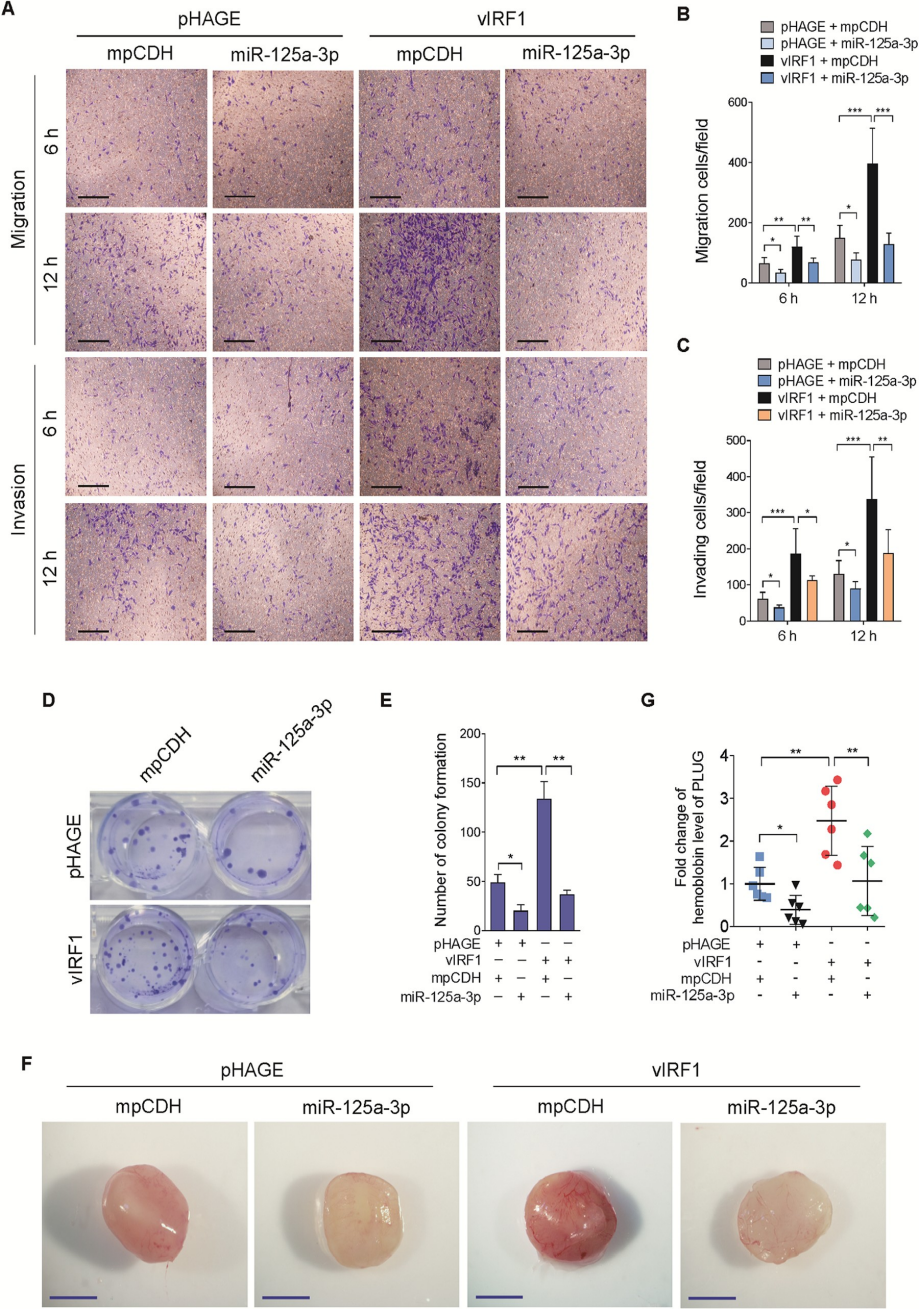
Overexpression of miR-125a-3p inhibits vIRF1-induced cell motility, proliferation and angiogenesis. **(A)**. Transwell migration (top) and Matrigel invasion (bottom) assays for pri-HUVECs transduced with lentiviral vIRF1 or its control pHAGE, which were subsequently co-transduced with lentivirus-miR-125a-3p (miR-125a-3p) and its control mpCDH. The representative images were captured at 6 and 12 h post seeding (original magnification, ×100). **(B)**. The quantification results of Transwell migration assay in (A). **(C)**. The quantification results of Matrigel invasion assay in (A). **(D)**. Plate colony formation assay of EA.hy926 cells treated as in (A). Plate colony formation assay was performed as described in the “Materials and methods” section. **(E)**. Quantification of cell colony formation described in (D). **(F)**. The mixture containing high concentration Matrigel and EA.hy926 cells treated as in (A) was injected into nude mice. Representational photographs of plugs were exhibited. Scar bars, 1 cm. **(G)**. The level of hemoglobin in plug tissues treated in (F) was measured by comparing the standard curve. Data were shown as mean ± SD. * *P* < 0.05, ** *P* < 0.01, and *** *P* < 0.001, *n*.*s*, not significant. Statistical significance was determined using one-way ANOVA followed by Tukey’s multiple comparisons test.

Next, we examined the effect of miR-125a-3p on vIRF1 induction of cell motility and angiogenesis. We found that overexpression of miR-125a-3p in vIRF1-transduced cells had an inhibitory effect on vIRF1-induced cell migration and invasion (Fig 5A–5C). Consistently, overexpression of miR-125a-3p impeded vIRF1-induced cell proliferation in a plate colony formation assay (Fig 5D and 5E). More importantly, induction of miR-125a-3p impaired vIRF1-enhanced angiogenesis *in vivo* based on a matrigel plug assay in mice (Fig 5F and 5G). Together these data suggest that circARFGEF1 binds to and degrades miR-125a-3p to promote vIRF1-induced cell invasion, proliferation and angiogenesis.

### miR-125a-3p directly targets GLRX3 to negatively regulate vIRF1-induced cell invasion and angiogenesis

**Fig 6:**
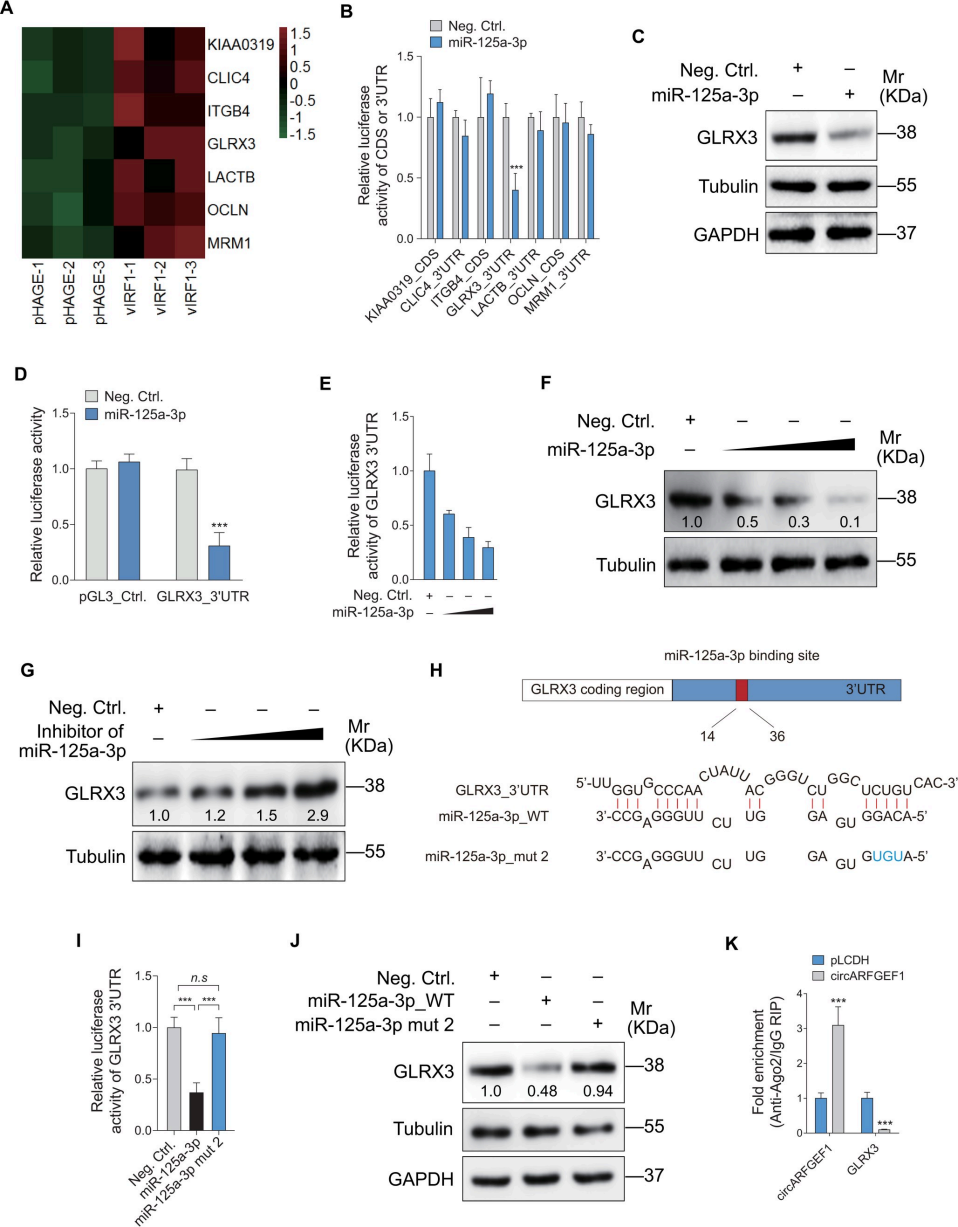
miR-125a-3p directly targets GLRX3. **(A)**. Heatmap analyses of mass spectrometry showing vIRF1 upregulated proteins that were predicted to be the targets of miR-125a-3p. **(B)**. miR-125a-3p mimic (miR-125a-3p, 20 nM) or its control (Neg. Ctrl.) were co-transfected into HEK293T cells with reporter plasmids for 48 h and then were used for luciferase activity. **(C)**. Western blotting of GLRX3 expression in EA.hy926 cells transfected with miR-125a-3p mimic (20 nM). **(D)**. Luciferase activity in HEK293T cells co-transfected with miR-125a-3p mimic (miR-125a-3p; 20 nM) or its control (Neg. Ctrl.) and the reporter construct GLRX3_3’UTR or its control reporter construct pGL3_Ctrl. for 48 h. **(E)**. Luciferase activity in HEK293T cells co-transfected with increasing amounts of miR-125a-3p mimic (miR-125a-3p; 10, 20, and 50 nM) or its control (Neg. Ctrl.), and the reporter construct GLRX3_3’UTR for 48 h. **(F)**. Western blotting of GLRX3 expression in EA.hy926 cells transfected with increasing amounts of miR-125a-3p mimic (10, 20 and 50 nM) or its control (Neg. Ctrl.) for 48 h. **(G)**. Western blotting of GLRX3 expression in EA.hy926 cells transfected with increasing amounts of miR-125a-3p inhibitor (10, 20 and 50 nM) or its control (Neg. Ctrl.) for 48 h. **(H)**. Putative binding site of miR-125a-3p in the 3’UTR region of GLRX3 and mutagenesis of target site in miR-125a-3p (miR-125a-3p mut 2). **(I)**. Luciferase activity in HEK293T cells co-transfected with miR-125a-3p mimic (20 nM), miR-125a-3p mutant 2 mimic (20 nM) or a negative control (Neg. Ctrl.) and the GLRX3_3’UTR reporter construct for 48 h. **(J)**. Western blotting of GLRX3 expression in EA.hy926 cells transfected with miR-125a-3p mimic (20 nM), miR-125a-3p mutant 2 mimic (20 nM) or a negative control (Neg. Ctrl.) for 48 h. **(K)**. RIP results in EA.hy926 cells showing the enrichment of binding of circARFGEF1, GLRX3 mRNA with miR-125a-3p based on Ago2 immunoprecipitation assay. Data were shown as mean ± SD. * *P* < 0.05, ** *P* < 0.01, and *** *P* < 0.001; *n*.*s*, not significant. Statistical significance was determined using Student’s t-test in B and K; one-way ANOVA followed by Tukey’s multiple comparisons test in D and I.

To identify the targets of miR-125a-3p, we examined proteins differential expressed between vIRF1- and pHAGE-transduced cells by mass spectrometry analysis (S1 Table). Bioinformatics analysis with several programs predicted the putative miR-125a-3p targets in vIRF1-upregulated proteins. As shown in Fig 6A, we predicted 7 proteins that might have miR-125a-3p putative binding sites in their CDS or 3’UTR. A luciferase reporter assay confirmed that only miR-125a-3p significantly decreased the luciferase activity of the 3’ UTR reporter of glutaredoxin 3 (GLRX3) (Fig 6B). Western blotting confirmed that miR-125a-3p inhibited GLRX3 protein expression (Fig 6C). miR-125a-3p suppressed the luciferase reporter activity of GLRX3 3’UTR in a dose-dependent manner (Fig 6D and 6E). Similarly, when EA.hy926 cells were transfected with increasing amounts of miR-125a-3p mimics or inhibitors, GLRX3 protein expression changed in a dose-dependent pattern (Figs 6F, 6G and S8). To identify the interacting site between miR-125a-3p and GLRX3 3’UTR, a miR-125a-3p mutant was generated based on BLAST prediction (Fig 6H). Mutation in seed sequence of miR-125a-3p dramatically eliminated the inhibitory effect of miR-125a-3p on GLRX3 expression (Fig 6I and 6J). Since Ago2 is a component of RNA-induced silencing complexes (RISC) which induce mRNA silencing [30], we employed Ago2-RIP assay to examine whether circARFGEF1 acts as competitive RNA (ceRNA) to abolish the function of miR-125a-3p by releasing its targets from Ago2-RISC. We found that binding of the GLRX3 transcript by the Ago2-RISC was abrogated when we overexpressed circARFGEF1 in EA.hy926 cells (Fig 6K). These results demonstrated that circARFGEF1 served as a miR-125a-3p sponge to reverse the inhibitory effect of miR-125a-3p on GLRX3.

**Fig 7:**
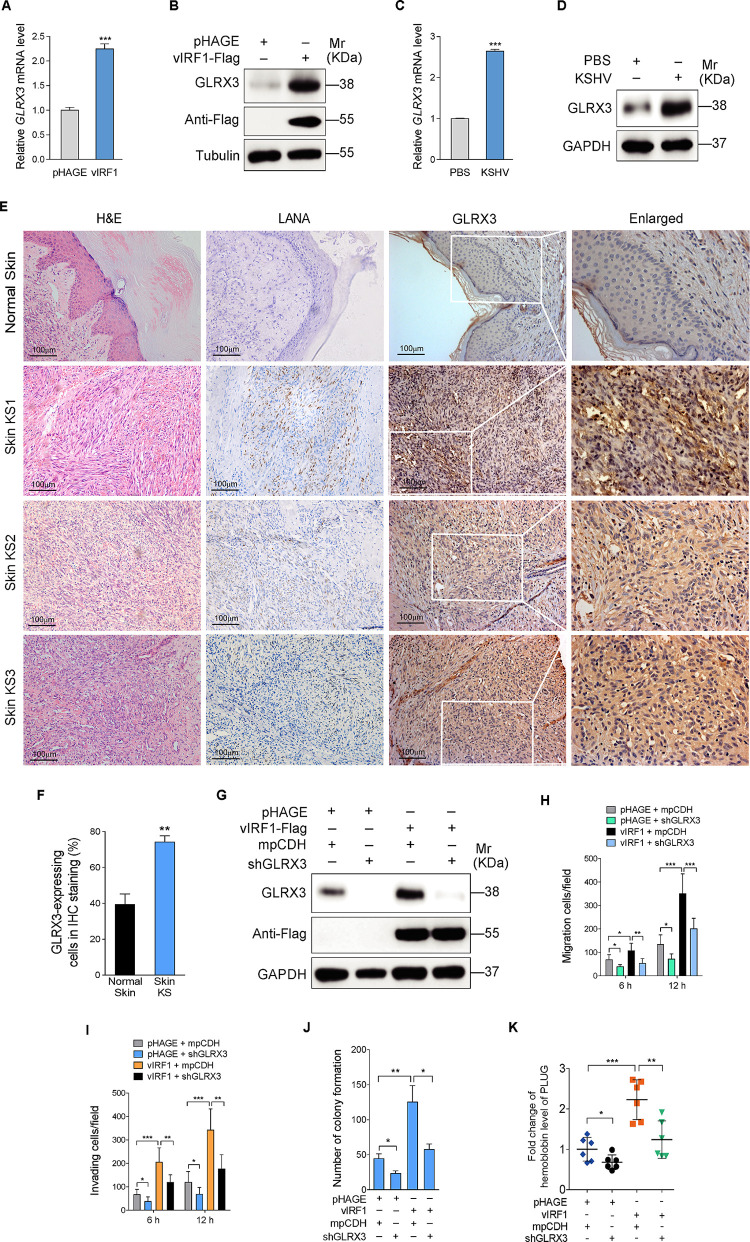
GLRX3 promotes vIRF1-induced cell motility, proliferation and angiogenesis. **(A)**. RT-qPCR analysis of GLRX3 mRNA level in vIRF1-transduced EA.hy926 cells or its control pHAGE-transduced cells. **(B)**. Western blotting analysis of GLRX3 protein level in vIRF1-transduced EA.hy926 cells or its control pHAGE-transduced cells. **(C)**. RT-qPCR analysis of GLRX3 mRNA level in KSHV (3 MOI) infected EA.hy926 cells or its control PBS-treated cells. **(D)**. Western blotting analysis of GLRX3 protein level in KSHV (3 MOI) infected EA.hy926 cells or its control PBS-treated cells. **(E).** H&E staining and immunohistochemical staining of KSHV LANA and GLRX3 in normal skin and skin KS of 3 patients. Magnification, ×200, ×400. **(F)**. Results were quantified in (E), data were shown as mean ± SEM, ** *P* < 0.01. The results were processed and analyzed using Image-Pro Plus 6.0 image analysis system (Media Cybernetics, Silver Spring, MD). Five random fields for each patient were chosen under the microscope and further measured for area and intensity of the expression of target protein, with the expression level of target protein calculated based on average absorbance (gray). **(G)**. Western blotting analysis of GLRX3 protein in vIRF1-expressing EA.hy926 cells transduced with a mixture of lentivirus-mediated shRNA targeting GLRX3 (shGLRX3). **(H)**. Migration analysis of pri-HUVECs treated as in (G). Transwell migration assay was performed as described in the “Materials and methods” section. **(I)**. Invasion analysis of pri-HUVECs treated as in (G). Matrigel invasion assay was performed as described in the “Materials and methods” section. **(J)**. Plate colony formation assay of EA.hy926 cells treated as in (G). Plate colony formation assay was performed as described in the “Materials and methods” section. **(K)**. The mixture containing high concentration Matrigel and EA.hy926 cells treated as in (G) was injected into nude mice. The level of hemoglobin in mice plug tissues was measured by comparing the standard curve. Data were shown as mean ± SD. * *P* < 0.05, ** *P* < 0.01, and *** *P* < 0.001; *n*.*s*, not significant. Statistical significance was determined using Student’s t-test in A, C and F; one-way ANOVA followed by Tukey’s multiple comparisons test in H, I, J and K.

To further explore the role of GLRX3 in vIRF1-induced pathogenesis, we examined GLRX3 expression level in vIRF1-transduced cells. RT-qPCR and Western blotting showed that both mRNA and protein levels of GLRX3 were elevated in vIRF1-transduced EA.hy926 cells (Fig 7A and 7B). Consistent with these observations, mRNA and protein levels of GLRX3 were also increased in KSHV-infected cells (Fig 7C and 7D). Immunohistochemistry (IHC) staining was performed to examine GLRX3 distributions in KS lesions. There were more GLRX3-positive cells in KS lesions compared to normal skin (Fig 7E and 7F). To determine whether upregulated GLRX3 is required for vIRF1 promoted cell motility and angiogenesis, we performed knockdown of GLRX3 with lentivirus-mediated shRNAs in vIRF1-transduced cells (Figs 7G and S9). We found that knockdown of GLRX3 decreased vIRF1-induced oncogenic phenotypes (Figs 7H–7K and S10). These data collectively suggest that miR-125a-3p directly targets GLRX3 to negatively regulate vIRF1-induced cell invasion, proliferation and angiogenesis.

### vIRF-circARFGEF1/miR-125a-3p/GLRX3 axis is essential for KSHV-induced cell invasion and angiogenesis

**Fig 8:**
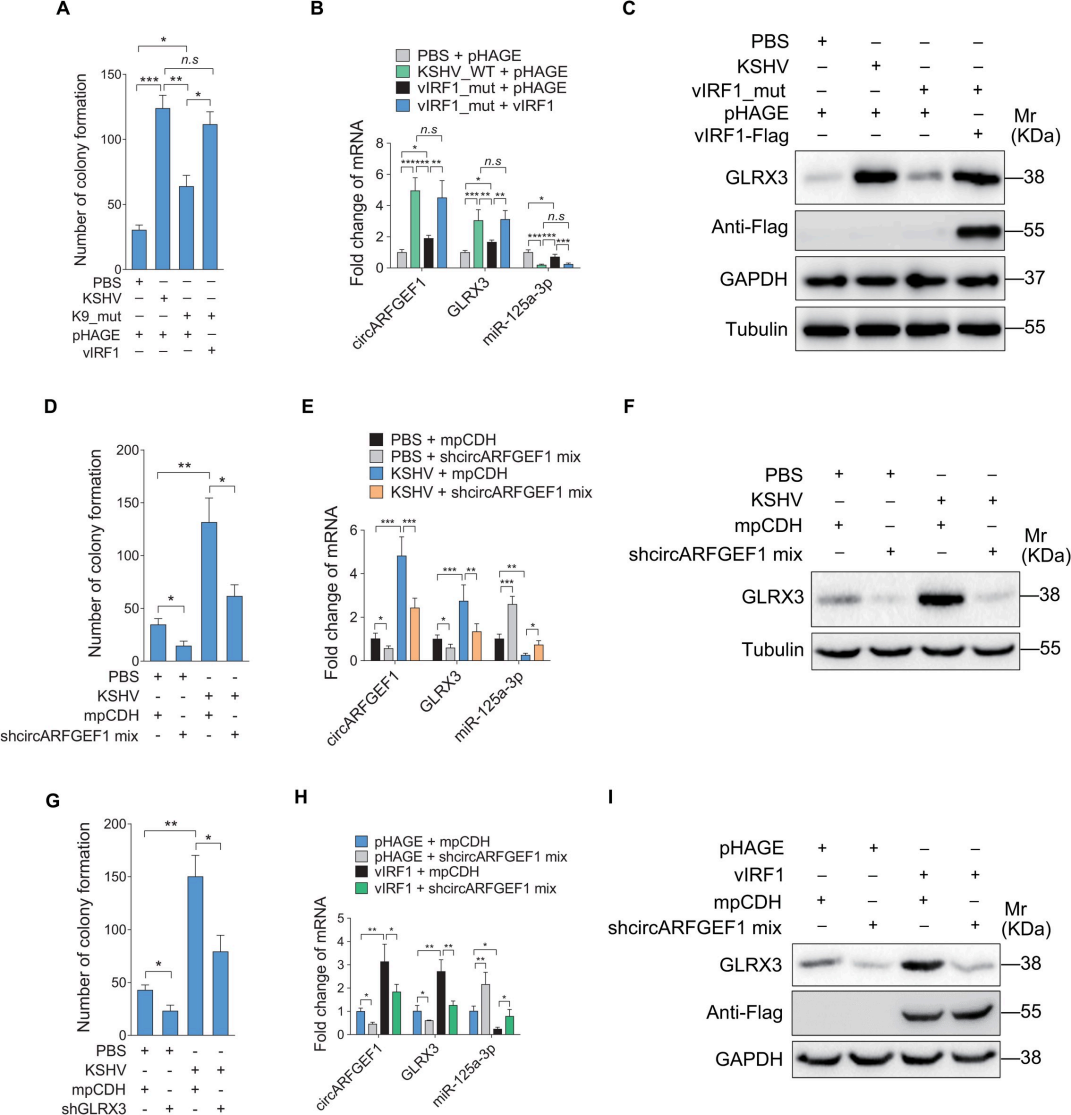
Loss of vIRF1 gene from KSHV genome reverses the tendency of circARFGEF1/miR-125a-3p/GLRX3 axis regulated by KSHV. **(A)**. Plate colony formation analysis of EA.hy926 cells treated with PBS (PBS), infected with KSHV wild type virus (KSHV_WT; 3 MOI), vIRF1 mutant virus (vIRF1_mut), or vIRF1 mutant virus followed by transduction with lentiviral vIRF1 at MOI 2. **(B)**. RT-qPCR analysis of levels of circARFGEF1, miR-125a-3p and GLRX3 mRNA in cells treated as in (A). **(C)**. Western blotting analysis of GLRX3 protein in cells treated as in (A). **(D)**. Plate colony formation analysis of EA.hy926 cells treated with PBS (PBS), infected with KSHV wild type virus (3 MOI) or transduced with lentivirus-mediated shcircARFGEF1 sequence targeting circARFGEF1. **(E)**. RT-qPCR analysis of levels of miR-125a-3p and GLRX3 mRNA in KSHV (3 MOI) infected EA.hy926 cells transduced with lentivirus-mediated shcircARFGEF1 or its control mpCDH. **(F)**. Western blotting analysis of the level of GLRX3 protein in cells treated as in (E). **(G)**. Plate colony formation analysis of EA.hy926 cells treated with PBS (PBS), infected with KSHV wild type virus (3 MOI) or transduced with lentivirus-mediated shGLRX3 targeting GLRX3. **(H)**. RT-qPCR analysis of levels of miR-125a-3p and GLRX3 mRNA in vIRF1-expressing EA.hy926 cells transduced with lentivirus-mediated shcircARFGEF1 or its control mpCDH. **(I)**. Western blotting analysis of the level of GLRX3 protein in cells treated as in (H). Data were shown as mean ± SD. * *P* < 0.05, ** *P* < 0.01, and *** *P* < 0.001, *n*.*s*, not significant. Statistical significance was determined using one-way ANOVA followed by Tukey’s multiple comparisons test.

To determine the role of circARFGEF1/miR-125a-3p/GLRX3 axis in vIRF1-induced cell invasion and angiogenesis in the presence of KSHV genome, we employed a KSHV mutant virus with deleted ORF-K9 to infect cells [16]. We have previously shown that deletion of vIRF1 from KSHV genome not only decreased KSHV-induced cell migration and invasion [16], but also lowered KSHV-induced angiogenesis and cellular transformation [9,17]. Here, we found that deletion of vIRF1 abolished the ability of KSHV-induced cell proliferation in a plate colony formation assay; however, complementation with a vIRF1 construct in the vIRF1_mut virus infected cells recovered this phenomenon (Fig 8A). Further, deletion of vIRF1 attenuated mRNA levels of circARFGEF1 and GLRX3, while complementation with the vIRF1 construct rescued their transcript levels (Fig 8B). Correspondingly, the mRNA level of miR-125a-3p had an inverse change compared to those of circARFGEF1 and GLRX3 mRNA (Fig 8B). Consistent with these observations, deletion of vIRF1 decreased GLRX3 protein level, which were completely reversed following vIRF1 complementation (Fig 8C). Additionally, silencing of circARFGEF1 in KSHV-infected cells not only reduced the ability of KSHV-induced cell proliferation in a plate colony formation assay (Figs 8D and S11A), but also resulted in increased miR-125a-3p and decreased GRLX3 (Fig 8E and 8F). Furthermore, knockdown of GLRX3 in KSHV-infected cells decreased their efficiency of plate colony formation (Figs 8G and S11B). Meanwhile, silencing of circARFGEF1 in vIRF1-transduced cells exhibited the similar results (Fig 8H and 8I). Collectively, these data indicate that by hijacking circARFGEF1 to function as a ceRNA, vIRF1 enhances cell motility, proliferation and angiogenesis by downregulating miR-125a-3p and releasing its target GLRX3.

## Discussion

Although KSHV vIRF1 was characterized as a lytic protein [8,11], low level of vIRF1 expression was detected in KSHV-infected cells in KS tumors and PEL cells [10,31,32], suggesting that its expression in tumors could have occurred in a subset of lytically infected cells. In fact, about 1–3% of the KSHV-infected cells in KS tumors express viral lytic proteins [33–36]. The low rate of spontaneous lytic replication is presumed to produce infectious virions to infect new cells. Both the low rate of spontaneous lytic replication and *de novo* infection of new cells with infectious virions contribute to KSHV-induced pathogenesis, including that KSHV oncogenic proteins drive cell invasion, angiogenesis, and cellular transformation. On the other hand, both virus-encoded cytokines and cellular cytokines, growth factors and other cellular proteins induced during the low rate of spontaneous lytic replication and *de novo* infection of new cells by infectious virions promote KS pathogenesis through autocrine and paracrine mechanisms. Consistently, our most recent study reported that vIRF1-positive cells could be observed in KS lesions, indicating that the expression of vIRF1 protein is most likely due to viral lytic replication [9]. Thus, vIRF1 expression in tumors reinforces its critical role in KS tumorigenesis. It has been shown that vIRF1 shares significant homology with cellular IRF-4, -8, -9 at the DNA binding domain (DBD) [37]. The crystal structure of the vIRF1 DBD also supports that vIRF1 is a DNA-binding protein [38]. Consistent with these findings, vIRF1 was found to locate in the promoters of KSHV ORF-K3, vDHFR and vIL-6, resulting in the induction of these genes [39]. Here, we firstly reported the vIRF1 binding to the promoter of cellular gene ARFGEF1 to activate its transcription through interaction with the transcriptional factor Lef1. Despite more pre-mRNA of ARFGEF1 was generated after ARFGEF1 transcription activation, the mRNA and protein levels of ARFGEF1 did not change, while circARFGEF1 was significantly increased in vIRF1-transduced cells and KSHV infected cells. It is possible that this is due to the competition between canonical pre-mRNA splicing and circularization of exons [40]. This led us to speculate that some circRNA-related splicing factors might be dysregulated by vIRF1 and KSHV, resulting in more circARFGER1 production rather than ARFGEF1. Therefore, these elusive molecules and mechanisms remain to be further explored.

Currently, mounting evidence indicates that circRNAs play indispensable roles in various diseases, especially in cancers [41]. Surprisingly, KSHV itself makes multiple circRNAs (circ-vIRF4, circK7.3s and circPANs) [23]. Circ-vIRF4 was highly expressed in BCBL-1 cells but was lowly expressed in BC1 cells [25], indicating the distinct circRNAs expression patterns in the different cells. Cytoplasmic circ-vIRF4 was a component of KSHV viral particles, and circPANs was associated with viral life cycle [25]. Besides circ-vIRF4 and circPANs, many other KSHV circRNAs were detected by circRNAs sequencing in BCBL-1 and BC1 cells [23]. Similarly, using primary human umbilical vein endothelial cells (pri-HUVECs) or a B cell line (MC116, KSHV negative), the cellular circRNAs profile was examined upon *de novo* infection with KSHV for 6 h using circRNA microarray [24]. A total of 264 and 285 dysregulated circRNAs were identified in KSHV-infected pri-HUVECs and MC116 cells, respectively, but only 7 of the same circRNAs were dysregulated in both cell types [24]. In the current study, we established the profile of circRNAs in KSHV-infected EA.hy926 cell line, which was generated by fusing primary human umbilical vein endothelial cells with a thioguanine-resistant clone of A549 (a human lung carcinoma cell line) in the presence of polyethylene glycol (PEG), and the hybrid clones were subsequently selected in hypoxanthine-aminopterin-thymidine (HAT) medium and screened for factor VIII-related antigen. We had compared our results to those of pri-HUVECs, however, we found only one circRNA that was dysregulated in both two cells. We speculated that this is probably due to the genetic heterogeneity and different methods of KSHV infection in these two types of cells. This scenario is similar to the differences of circRNAs between KSHV-infected pri-HUVECs and MC116 [24]. Although KSHV-regulated cellular circRNAs have been identified and KSHV decreased hsa_circ_0001400 regulates the expression of KSHV genes (*LANA* and *RTA*) and cell growth [24], vIRF1-regulated cellular circRNAs remains unclear. Here we identified circARFGEF1 as a circRNA upregulated in both vIRF1-transduced and KSHV-infected cells. Mechanistically, vIRF1 enhanced circARFGEF1 transcription, potentially by forming a complex with transcription factor Lef1, resulting in cell migration, invasion, proliferation, and *in vivo* angiogenesis. circARFGEF1 was the first reported circRNA regulated by a KSHV gene and it mediated KSHV induction of oncogenic phenotypes. Of course, our results did not exclude the possibility that other KSHV genes could also regulate circRNAs and that vIRF1 might also regulate other circRNAs involved in KSHV-induced oncogenesis.

To search for the downstream miRNAs regulated by circARFGEF1, we re-analyzed our previous data of vIRF1-regulated miRNA array [16]. miR-125a-3p, which was generated from hsa-miR-125a Stem-loop sequence (Accession ID: MI0000469), was a down-regulated miRNA in both vIRF1-transduced and KSHV-infected cells. miR-125a-3p exhibits a tumor suppressive function in most tumors, such as gliomas, hepatocellular carcinoma, renal cell carcinoma and ovarian carcinoma [42–45]. Similar to miR-125a-3p, miR-125a-5p was decreased in gliomas, breast cancer, lung adenocarcinoma and ovarian cancer [46–49]. In contrast, miR-125a-3p was significantly up-regulated in early lung adenocarcinomas [50]. miR-125a-3p inhibited renal cell angiogenesis by targeting VEGF [44]. In gliomas, circ-MAPK4 inhibited cell apoptosis and promoted cell proliferation via activating p38/MAPK signaling by sponging miR-125a-3p in gliomas [42]. In addition, lncRNA MALAT1 modulated miR-125a-3p to increase FOXM1 expression, resulting in hepatocellular carcinoma progression [43]. Similar to most tumors, in this study, our results also indicated a tumor suppressive function of miR-125a-3p. miR-125a-3p was decreased in both KSHV-infected cells and vIRF1-transduced cells, and overexpression of miR-125a-3p reversed vIRF1-induced oncogenic phenotypes. Furthermore, circARFGEF1 served as a sponge and bound to miR-125a-3p resulting in its decreased stability. One mechanism that mediates destabilization of miRNAs is through RNA-directed miRNA degradation (TDMD). Artificial targets with extensive complementarity to the miRNA can trigger TDMD through a poorly understood process associated with tailing (addition of untemplated nucleotides) and trimming of the miRNA 3′ terminus [51,52]. However, whether vIRF1 decreases miR-125a-3p level through its precursor is still unknown.

Although GLRX3, also known as protein kinase C-interacting cousin of thioredoxin (PIOCT), has been identified for almost two decades, its biological function remains largely unclear. GLRX3 was recently reported to bind to polycomb group (PcG) proteins resulting in enhanced methylation of PcG target gene promoter [53,54]. Moreover, GLRX3 was an important factor for regulating stress-induced DNA-damage response. In oral squamous cell carcinoma, upregulated GLRX3 promoted cell migration and invasion through the Notch signaling [55]. In this study, we have identified GLRX3 as a direct target of miR-125a-3p. GLRX3 was increased in vIRF1-transduced cells, KSHV-infected cells, and KS lesions. Knockdown of GLRX3 reversed vIRF1-induced cell motility, proliferation and *in vivo* angiogenesis, indicating that it has the characteristics of tumor genes. However, how GLRX3 promotes vIRF1-induced cell motility, proliferation and angiogenesis remains to be further explored.

**Fig 9:**
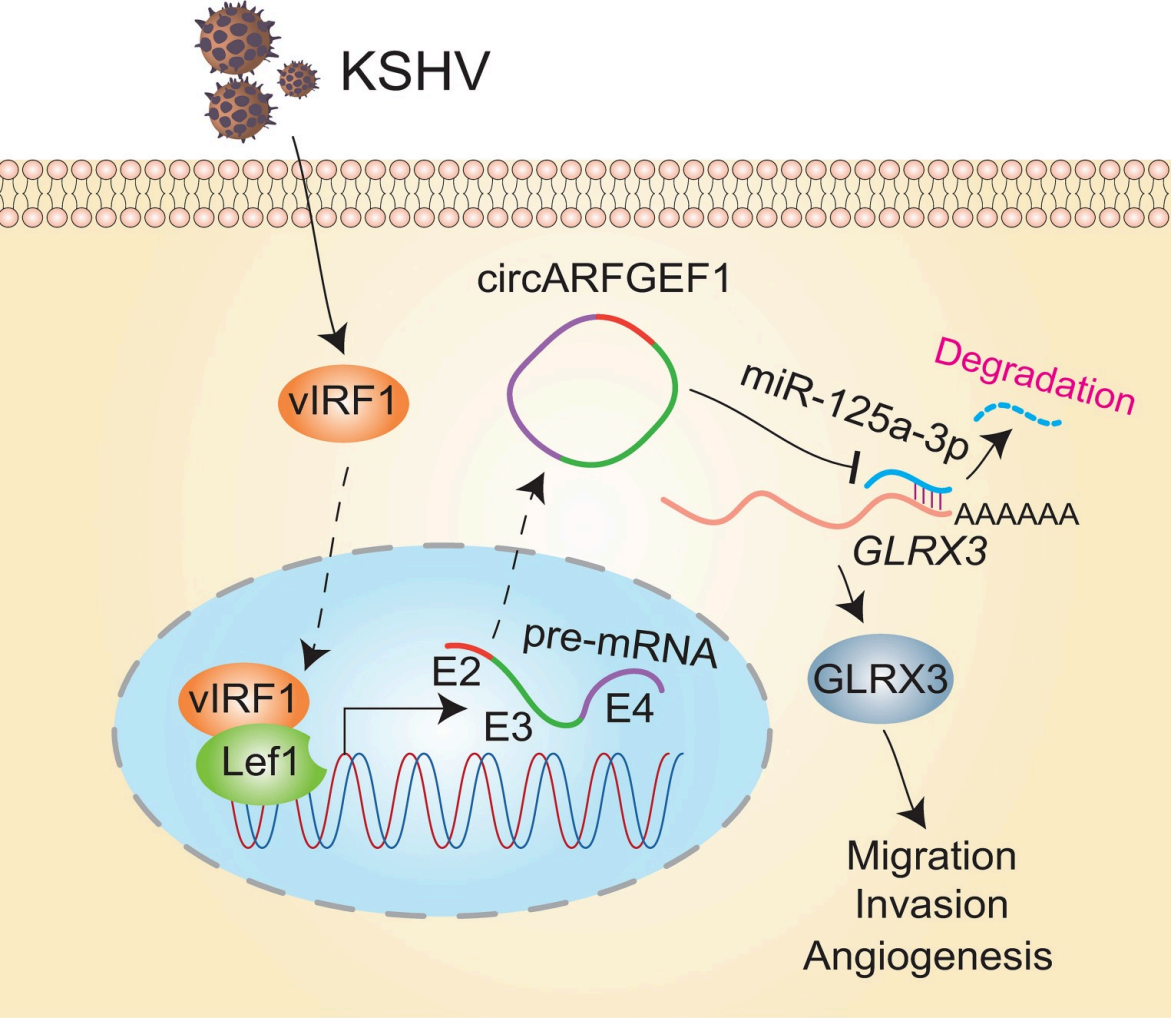
A hypothetical model of the mechanism of how vIRF1 regulates circARFGEF1/miR-125a-3p/GLRX3 axis. vIRF1 transcriptionally activates circARFGEF1 through interacting with Lef1, leading to the instability of miR-125a-3p, and increasing of GLRX3. Upregulation of GLRX3 protein promotes vIRF1 induction of cell migration, invasion and angiogenesis.

In this study, we focused on the mechanism by which vIRF1 participates in KSHV-induced tumorigenesis by regulating cellular noncoding RNA. vIRF1 transcriptionally activates circARFGEF1 through interacting with Lef1, leading to the instability of miR-125a-3p, and increasing of GLRX3 (Fig 9). Our novel findings shed light on the regulatory network of cellular circRNA and its downstream pathway involved in KS pathogenesis, providing potential therapeutic targets of KSHV-induced cancers. This work also points to a new direction for understanding and exploring the pathogenesis of cancers induced by oncogenic viruses.

## Materials and methods

### Ethics statement

The KS clinical specimens were collected from the First Affiliated Hospital of Nanjing Medical University. The protocol was reviewed and ethically approved by the Institutional Ethics Committee of the First Affiliated Hospital of Nanjing Medical University (Nanjing, China). Written informed consent was obtained from all anonymized adult participants.

### Cells and transfection

iSLK-RGB-BAC16 and iSLK-RGB-K9 mutant cells were grown in DMEM supplemented with 10% fetal bovine serum (FBS), 1 μg/ml puromycin, 250 μg/ml G418, and 1.2 mg/ml hygromycin B. Primary human umbilical vein endothelial cells (pri-HUVECs), which were used between passages 3 and 6, were isolated from the interior of the umbilical vein of human umbilical cords by digestion with collagenase (Sigma, St. Louis, MO, USA), and cultured in complete EBM-2 culture media (LONZA, Allendale, NJ, USA) as previously described [56]. pri-HUVECs were used for migration and invasion assays, a human umbilical vein endothelial cell line, EA.hy926 (catalog #CRL-2922; ATCC, Manassas, VA, USA) was employed for RNA-seq analysis, plate colony formation assay and *in vivo* matrigel plug assay, and HEK293T cells were used for lentivirus packaging and luciferase activity assay. Both HEK293T and EA.hy926 were maintained in DMEM supplemented with 10% fetal bovine serum (FBS). All of cell lines were authenticated by short tandem repeat profiling. Effectence transfection reagent (Qiagen, Suzhou, Jiangsu, China) and Lipofectamine 2000 Reagent (Invitrogen, Carlsbad, CA, USA) were used for the transfection of endothelial cells and HEK293T cells, respectively.

### Plasmids, lentivirus packaging and infection

The sequence of circARFGEF1 promoter was amplified from pri-HUVECs according Genecopoeia annotation and cloned into pGL3-basic plasmid (Promega, Shanghai, China). The pGL3-control vector was used to generate pGL3-circARFGEF1 and pGL3-GLRX3_3’UTR constructs by inserting the indicating sequences to the downstream of luciferase gene. The construct pGL3-circARFGEF1 contained full length of circARFGEF1 cDNA sequence, and pGL3-GLRX3_3’UTR included the full length of GLRX3 3’UTR sequence. Lef1 encoding sequences with HA tag at c-terminus were amplified by RT-PCR and cloned into pCDNA3.1 vector. The full-length of circARFGEF1 cDNA was amplified and cloned into the circRNA expression vector pLCDH-ciR (Geneseed Biotech Co, China), which contains front and back circular flanking sequences. microRNA mimics and related mimics control were got from GenePharma (Shanghai, China). The miR-125a-3p expressing plasmid was generated as previously described [16]. Briefly, lentiviral vector pCDH-CMV-MCS-EF1-copGFP was modified to contain miR-30 precursor stem-loops. Precursor stem-loops of miR-125a-3p was synthesized and cloned into modified pCDH (mpCDH). Similarly, short hairpin RNA (shRNA) expressing vectors were generated using mpCDH plasmid as previously described [57]. The shRNAs target the circARFGEF1 back-splice junction so as not to influence linear ARFGEF1. The shRNA sequences of circARFGEF1, Lef1 and GLRX3 were listed in S2 Table.

Lentivirus packaging and infection of target cells were performed as previously described [58]. Briefly, the packaging plasmid, psPAX2, together with the envelope plasmid, pMD2.G, and the recombinant lentivirus plasmids were co-transfected into pre-inoculated HEK293T cells. Lentivirus was collected and used to infect cells. The infection efficiency was monitored at 48 to 72 h post infection, and the infected cells were collected at the indicated time points for further analyses.

### Generation of KSHV_WT and vIRF1_mutant recombinant virus

KSHV_WT and vIRF1_mutant constructs were previously described [16]. iSLK-RGB-BAC16 and iSLK-RGB-K9 mutant cells were used for the generation of KSHV_WT virus and vIRF1_mutant virus, which were induced by sodium butyrate and doxycycline as previously described [59]. By incubating 1×10^7^ iSLK-RGB-BAC16 and iSLK-RGB-K9 mutant cells with Doxycycline (Dox) (1 μg/ml) and sodium butyrate (NaB) (1 mM) for 4–5 day, the supernatant was collected by ultracentrifugation (25, 000 g at 4°C for 3 h) using SW32 Ti rotor (Beckman Coulter Inc, USA). The virus pellet was resuspended by DMEM supplemented with 8 μg/mL polybrene and then was subjected to infect 10^5^ indicated cells in 6 well plate at MOI of 3.

### Expression profile analysis of circRNAs

Total RNAs were extracted from 1×10^7^ cells using TRIzol regeant. The RNA quality (purity and concentration) was examined by NanoDrop ND-1000 (Thermo Fisher Scientific, USA). The ribosomal RNA was removed using Ribo zero rRNA removal Kit (Ribobio, China). After fragmenting to approximately 200 bp, 1^st^ and 2^nd^ strand cDNA was synthesized according to the instructions of NEBNext Ultra RNA Library Prep Kit for Illumina (NEB, USA) and then sequenced on HiSeq 3000 with 2 × 150 bp mode. Two algorithms, CIRI2 and CIRCexplorer2 were used to detect circRNAs. Reads were mapped to human reference genome GRCh37/ hg19 (http://genome.ucsc.edu/) by BWA-MEM or Tophat, respectively. If a circRNA can be detected by both methods, it will be considered as an identified cirRNA. Back-spliced junction reads identified in CIRI2 were combined and scaled to RPM (Reads Per Million mapped reads, bwamem mapping) to quantify every circRNAs. RNA-seq data have been submitted and can be accessed by GEO accession number GSE157615.

### RT-PCR and qPCR

Total RNAs were transcribed into cDNA using HiScript III 1st Strand cDNA Synthesis Kit (+gDNA wiper) (Vazyme Biotech, China), according to manufacturer’s instructions. Target sequence of circARFGEF1 was amplified using 2 × Phanta PCR Mix (Vazyme Biotech, China) following manufacturer’s instructions. RT-qPCR was employed to detect the target mRNAs or miR-125a-3p. GAPDH, Actin or U6 was served as the internal control to normalize the qPCR results. The PCR and RT-qPCR primers were listed in S3 Table.

### RNA-seq analysis

The RNA-seq analysis was performed by Guangzhou RiboBio Co., Ltd. Briefly, RiboMinus Eukaryote Kit (Qiagen, CA) was used to remove ribosomal RNA; NEBNext Ultra Directional RNA Library Prep Kit for Illumina (NEB, USA) was employed to prepare strand-specific RNA-seq libraries; the cDNA products were sequenced in a HiSeq 2000 system (Illumina, USA).

### RNA preparation and treatment with RNase R or actinomycin D

Total RNAs were extracted from indicated cells using TRIzol RNA Isolation Reagents (Life Technologies, Grand Island, NY, USA), according to manufacturer’s instructions. Then 2 μg RNAs were incubated with 6 U RNase R (Epicenter Technologies, USA) for 20 min at 37°C. For RNA degradation analysis, 2 mg/mL actinomycin D (Sigma-Aldrich, Germany) was added into cell cultured medium for indicated time.

### Western blotting and antibodies

Western blotting was performed as previously described [60]. Cells were harvest by ice-cold RIPA lysis buffer, and an equal amount of protein was loaded into 10% SDS-PAGE gel. After electrophoresis, the gel was transferred into 0.45 μm polyvinylidene difluoride (PVDF) membrane. After blocking with 5% non-fat milk, the membranes were incubated with primary antibodies: anti-GLRX3 (ABclonal,[25]anti-Flag (Cell Signaling Technology, USA). Horseradish Peroxidase (HRP) conjugated secondary antibodies were used to detect the signals.

### Immunohistochemistry (IHC)

The KS lesions and normal skin tissues were fixed in formalin. Tissues were embedded in paraffin and cut in a microtome to 5 μm. After deparaffinization, the antigen was retrieved using sodium citrate buffer. Then the tissues were incubated with GLRX3 antibody at 1: 100 dilution (ABclonal, China). HRP-labeled Goat-Anti-Rabbit secondary antibody was used to detect the signals.

### Dual-luciferase reporter assay

Dual-luciferase reporter assay was performed using the Dual-Luciferase Reporter (DLR) Assay System (Promega, USA), according to manufacturer’s instructions and our previous study [61–63]. Briefly, HEK293T cells were co-transfected with luciferase reporter plasmid and indicating gene plasmids for 48 h. The luminescence intensity of renilla and firefly luciferases was measured by a Luminometer.

### RNA immunoprecipitation (RIP) assay

RIP assay was performed as previously described [16]. Cells were transduced with lentiviral circARFGEF1 and its control pLCDH. The EZ-Magna RIP RNA-Binding Protein Immunoprecipitation Kit (Millipore, Germany) and anti-Ago2 antibody (Millipore, Germany) were used to examine mRNA levels of circARFGEF1 and GLRX3 on Ago2-RISC, according to the manufacturer’s instructions.

### RNA pull-down assay

RNA pull-down assay was performed as previously described by using biotin labeled miR-125a-3p probes [16]. Briefly, the cells were harvest by lysis buffer (Thermo Fisher Scientific, USA), and then incubated with streptavidin magnetic beads (Thermo Fisher Scientific, USA) and Biotin-labeled oligos. RT-qPCR was employed to detect the binding RNAs.

### Mass spectrometry

Mass spectrometry analysis was performed as previously delineated [56, 60]. Briefly, proteins were reduced, alkylated, and digested with trypsin. The resulting peptides were labeled with isobaric tandem mass tags (TMT 6-plex; Thermo Fisher Scientific Inc., San Jose, CA), mixed, and fractionated by strongcation exchange chromatography. Peptide digests were analyzed by nanoscalereversed phase liquid chromatography (Easy-nLC, Thermo Fisher Scientific Inc.) coupled online with an LTQ-OrbitrapVelos mass spectrometer (Thermo Fisher Scientific Inc.). Spectra were searched using Maxquant (version 1.2.2.5), and results were filtered to 1% FDR at the unique peptide level using the COMPASS software suite.

### Transwell migration and Matrigel invasion assays

Transwell migration and Matrigel invasion were performed as previously described [57,64]. Briefly, 1 × 10^5^ pri-HUVECs were seeded into 8 μm chambers (8-μm-pore polyethylene terephthalate membrane filter, Merck Millipore, Darmstadt, Germany) with or without matrigel coated, and were harvested 6 or 12 h after incubation. The migrated cells were fixed, stained, photographed and calculated by counting stained cells in a double-blinded manner by two observers.

### Plate colony formation assay

To evaluate the ability of cell proliferation, the plate colony formation assay was performed as previously described [58]. Briefly, cells (about 2 × 10^2^) were seeded in each well of 12-well plates with complete medium. Cultures were supplemented with complete medium per week. Cells were then fixed in methanol/glacial acetic acid (7:1), washed with water and stained with 0.5% crystal violet. Colonies were scored 14–21 days after seeding the cells.

### Matrigel plug assay for angiogenesis in nude mice

Matrigel plug assay for angiogenesis in nude mice was performed as previously described [65–67]. Briefly, 3–4-week old male athymic BALB/c nu/nu mice were purchased from the Charles River laboratories and maintained under pathogen-free conditions. The mice experimental protocols were approved by Nanjing Medical University Experimental Animal Welfare Ethics Committee. The treated cells were harvested at subconfluence, washed with phosphate-buffered saline (PBS) and resuspended in serum-free medium. Cell aliquots (0.2 ml) were mixed with 0.4 ml of High Concentration Matrigel (BD Biosciences), and the mixture was immediately injected subcutaneously (s.c.) into the left flanks of nude mice. At day 10 after the injection, the mice were sacrificed, and the Matrigel plugs were removed from the mice. Small pieces of tissue were cut out and weighed, which were processed using the Drabkin’s reagent kit (Sigma–Aldrich, St Louis, MO, USA) with spectrophotometric analysis at 540 nm, followed by calculation of hemoglobin content based on the standard curve obtained.

### Chromosome immunoprecipitation (ChIP)

ChIP assay was performed following the manufacturer’s instructions. Briefly, the anti-Flag and Lef1 antibodies immunoprecipitated DNAs were obtained by eluting the magnetic beads with ChIP elution buffer, and were purified for analysis using the circARFGEF1 promoter-specific primers through RT-qPCR. The sequences of circARFGEF1 promoter primers were provided in S2 Table.

### Statistics

All data were presented with mean ± SD except the IHC results were presented with mean ± SEM. All experiments were performed at least three times, unless otherwise stated. The results were analyzed by Student’s *t* test. ANOVA followed by a pairwise test was used for multiple comparisons when examine more than 2 conditions. We consider values * *P* < 0.05, ** *P* < 0.01, and *** *P* < 0.001 significant.

## Supporting information

S1 Fig Determination of transduction efficiency of lentivirus-mediated vIRF1 in endothelial cells. qPCR results showing vIRF1 mRNA expression in EA.hy926 cells infected with KSHV (3 MOI) or transduced with different MOI of lentiviral vIRF1. The level of vIRF1 mRNA in KSHV infected cells was set as “1” for comparison. The quantified results represent the mean ± SD.
(TIF)

S2 Fig The primer location of pre-mRNA and mature mRNA of ARFGEF1. The red lines indicate the locations of forward and reverse primers for pre-mRNA of ARFGEF1, and the blues ones indicate the locations of primers for mature mRNA of ARFGEF1.
(TIF)

S3 Fig Neither KSHV infection nor ectopic expression of vIRF1 affects ARFGEF1 protein expression. **(A)**. ARFGEF1 was detected after ectopic expression of vIRF1 in EA.hy926 cells via Western blotting. **(B)**. ARFGEF1 was examined after KSHV (3 MOI) infection in EA.hy926 cells via Western blotting.
(TIF)

S4 Fig Overexpression of circARFGEF1 does not affect mRNA level of its parental gene ARFGEF1. **(A).** qPCR results showing circARFGEF1 expression in EA.hy926 cells infected with KSHV or transduced with different MOI of lentiviral circARFGEF1. The level of circARFGEF1 in KSHV cells was set as “1” for comparison. **(B).** qPCR results of circARFGEF1 and mRNA of its parental gene ARFGEF1 in EA.hy926 cells transduced with lentiviral circARFGEF1 at 1 or 4 MOI and its control pLCDH. Data were shown as mean ± SD. *** *P* < 0.001, Student’s t-test. *n*.*s*, not significant.
(TIF)

S5 Fig Knockdown of circARFGEF1 does not affect mRNA level of its parental gene ARFGEF1. **(A)**. circARFGEF1 was silenced by lentivirus-mediated two shRNAs targeting circARFGEF1 and the knockdown efficiency of circARFGEF1 was measured by qPCR. **(B).** circARFGEF1 and mRNA level of its parental gene ARFGEF1 in EA.hy926 cells transduced with lentivirus-mediated shRNAs mix targeting circARFGEF1 or the control mpCDH. Data were shown as mean ± SD. * *P* < 0.05; *** *P* < 0.001, Student’s t-test. *n*.*s*, not significant.
(TIF)

S6 Fig The representative images of vIRF1-induced cell motility with knockdown of circARFGEF1. Lentiviral vIRF1 transduced pri-HUVECs were further transduced with a mixture of shRNAs targeting circARFGEF1 (shcircARFGEF1 mix), and were subjected to Transwell migration and invasion assays described in the “Materials and methods” section. The migrated and invaded cells were counted at 6 h and 12 h post seeding. Representational photographs of migration and invasion were exhibited (original magnification, ×100). Quantification of Transwell migration and invasion assay was described in **Fig 3B** and **3C**.
(TIF)

S7 Fig The inhibitory cell colony formation by knockdown of circARFGEF1 is reversed by overexpression of circARFGEF1. EA.hy926 cells were transduced with lentivirus-mediated shcircARFGEF1 mix targeting circARFGEF1, and further transduced with lentivirus-mediated overexpression of circARFGEF1. Then cells were subjected to cell plate colony formation, which was fully described in “Materials and Methods” section. * *P* < 0.05, Statistical significance was determined using one-way ANOVA followed by Tukey’s multiple comparisons test.
(TIF)

S8 Fig miR-125a-3p inhibits GLRX3 protein expression in a dose-dependent manner. GLRX3 protein expression in EA.hy926 cells transfected with increasing amounts of miR-125a-3p mimic (10, 20 and 50 nM) or its control (Neg. Ctrl.) for 48 h was quantified in **Fig 6F**. The difference of GLRX3 reduction was analyzed for three independent experiments. *** *P* < 0.001, Student’s t-test.
(TIF)

S9 Fig Knock down of GLRX3 by shRNAs. Western blotting was performed with the indicated antibodies in EA.hy926 cells transduced with lentiviruses containing shRNA 1 and 2, and a mixture of the two shRNAs targeting GLRX3 or the control mpCDH. Experiments were independently repeated three times with similar results. Results shown were from a representative experiment.
(TIF)

S10 Fig The representative images of vIRF1-induced cell motility, plate colony formation and *in vivo* angiogenesis with knockdown of GLRX3. **(A).** GLRX3 was interfered by two different shRNAs in vIRF1 transduced pri-HUVECs. Cells were subjected to Transwell migration and invasion assay described in the “Materials and methods” section. The migrated and invaded cells were counted at 6 h and 12 h post seeding. Representational photographs of migration and invasion were exhibited (original magnification, ×100). Quantification of Transwell migration and invasion assay was described in **Fig 7H** and **7I**. **(B).** Plate colony formation assay of EA.hy926 cells treated as in (**A**) was performed as described in the “Materials and methods” section. Representational photographs of plate colony were exhibited. Quantification of plate colony formation assay was described in **Fig 7J**. **(C).** The mixture containing high concentration Matrigel and EA.hy926 cells treated as in (**A**) was injected into nude mice. The details were shown in the “Materials and methods” section. Representational photographs of plugs were exhibited. Scar bars, 1 cm. Quantification of hemoglobin in plug tissues was described in **Fig 7K**.
(TIF)

S11 Fig The representative images of KSHV-induced plate colony formation with knockdown of circARFGEF1 or GLRX3. **(A).** Plate colony formation analysis of EA.hy926 cells treated with PBS (PBS), infected with KSHV wild type virus (3 MOI) or transduced with lentivirus-mediated shcircARFGEF1 sequences targeting circARFGEF1. Plate colony formation assay was performed as described in the “Materials and methods” section. Quantification of plate colony formation assay was described in **Fig 8D**. **(B)**. Plate colony formation analysis of EA.hy926 cells treated with PBS (PBS), infected with KSHV wild type virus (3 MOI) or transduced with lentivirus-mediated shGLRX3 targeting GLRX3. Plate colony formation assay was performed as described in the “Materials and methods” section. Quantification of plate colony formation assay was described in **Fig 8G**.
(TIF)

S1 Table The cellular proteins dysregulated >1.5 folds in HUVECs expressing vIRF1. All dysregulated >1.5 folds proteins in HUVECs expressing vIRF1 were listed in this table including previously published ones (Li W et al. PLoS Pathog. 2019 Jan 30;15(1):e1007578).
(XLSX)

S2 Table The sequences of the shRNAs. (DOCX)

S3 Table The sequences of specific primers of RT-qPCR. (DOCX)
