# Correction: CircRNA ARFGEF1 functions as a ceRNA to promote oncogenic KSHV-encoded viral interferon regulatory factor induction of cell invasion and angiogenesis by upregulating glutaredoxin 3

**DOI:** 10.1371/journal.ppat.1014267

**Published:** 2026-05-27

**Authors:** Shuihong Yao, Xuemei Jia, Fei Wang, Liuxue Sheng, Pengxia Song, Yanhui Cao, Hongjuan Shi, Weifei Fan, Xiangya Ding, Shou-Jiang Gao, Chun Lu

S10 Fig was uploaded incorrectly. Please see the correct S10 Fig here.

## Supporting information

S10 Fig The representative images of vIRF1-induced cell motility, plate colony formation and in vivo angiogenesis with knockdown of GLRX3. **(A).** GLRX3 was interfered by two different shRNAs in vIRF1 transduced pri-HUVECs. Cells were subjected to Transwell migration and invasion assay described in the “Materials and methods” section. The migrated and invaded cells were counted at 6 h and 12 h post seeding. Representational photographs of migration and invasion were exhibited (original magnification, × 100). Quantification of Transwell migration and invasion assay was described in **Fig 7H** and **7I**. **(B).** Plate colony formation assay of EA.hy926 cells treated as in (**A**) was performed as described in the “Materials and methods” section. Representational photographs of plate colony were exhibited. Quantification of plate colony formation assay was described in **Fig 7J**. **(C).** The mixture containing high concentration Matrigel and EA.hy926 cells treated as in (**A**) was injected into nude mice. The details were shown in the “Materials and methods” section. Representational photographs of plugs were exhibited. Scar bars, 1 cm. Quantification of hemoglobin in plug tissues was described in **Fig 7K**.
(EPS)
