# Supplementary figures and images for: CircRNA ARFGEF1 functions as a ceRNA to promote oncogenic KSHV-encoded viral interferon regulatory factor induction of cell invasion and angiogenesis by upregulating glutaredoxin 3
Source: PLoS Pathog. 2021 Feb 4;17(2):e1009294. doi: 10.1371/journal.ppat.1009294 (PMC7888650; doi:10.1371/journal.ppat.1009294)

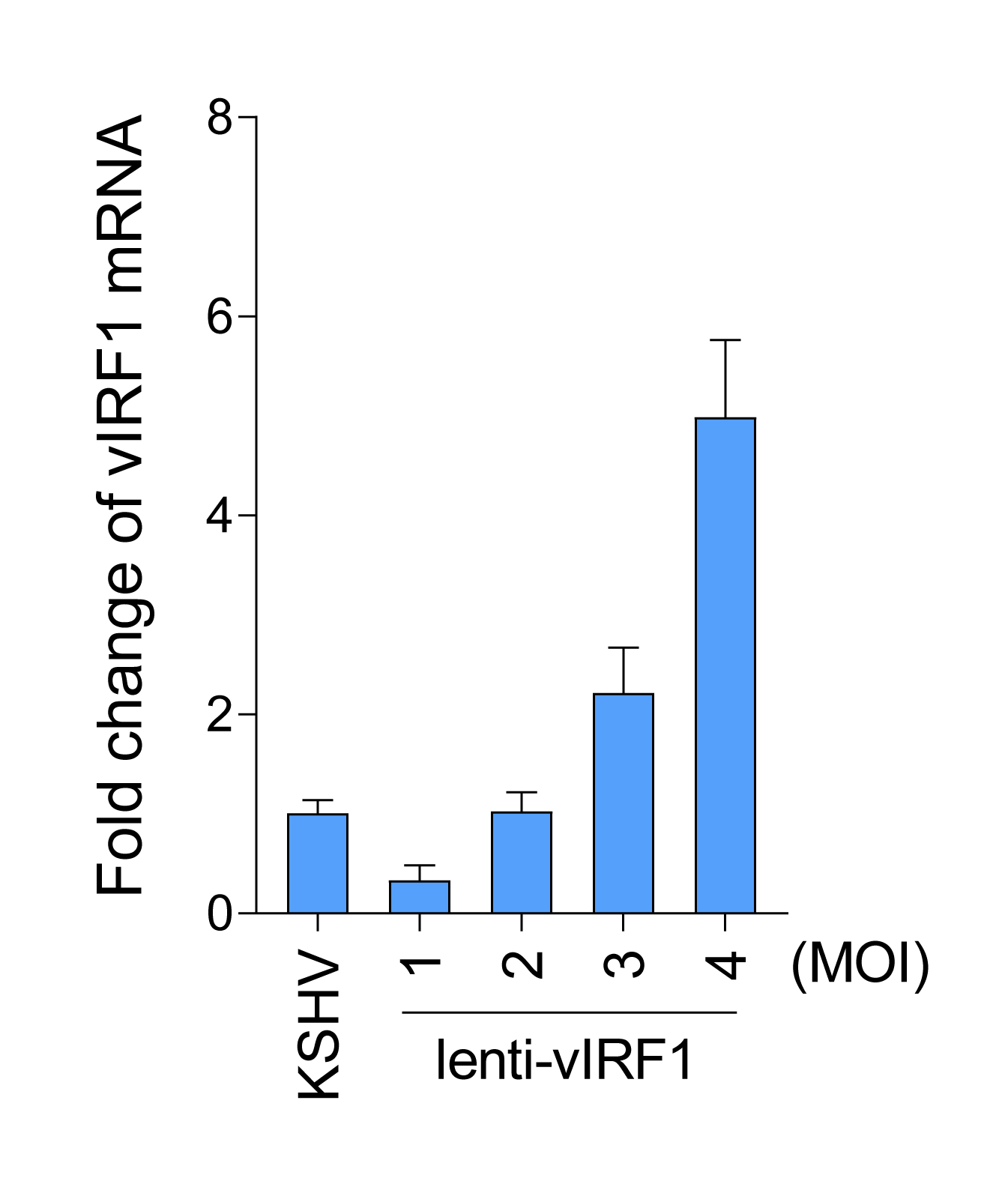

Supplement: S1 Fig — qPCR results showing vIRF1 mRNA expression in EA.hy926 cells infected with KSHV (3 MOI) or transduced with different MOI of lentiviral vIRF1. The level of vIRF1 mRNA in KSHV infected cells was set as “1” for comparison. The quantified results represent the mean ± SD. (TIF) [file ppat.1009294.s001.tif]

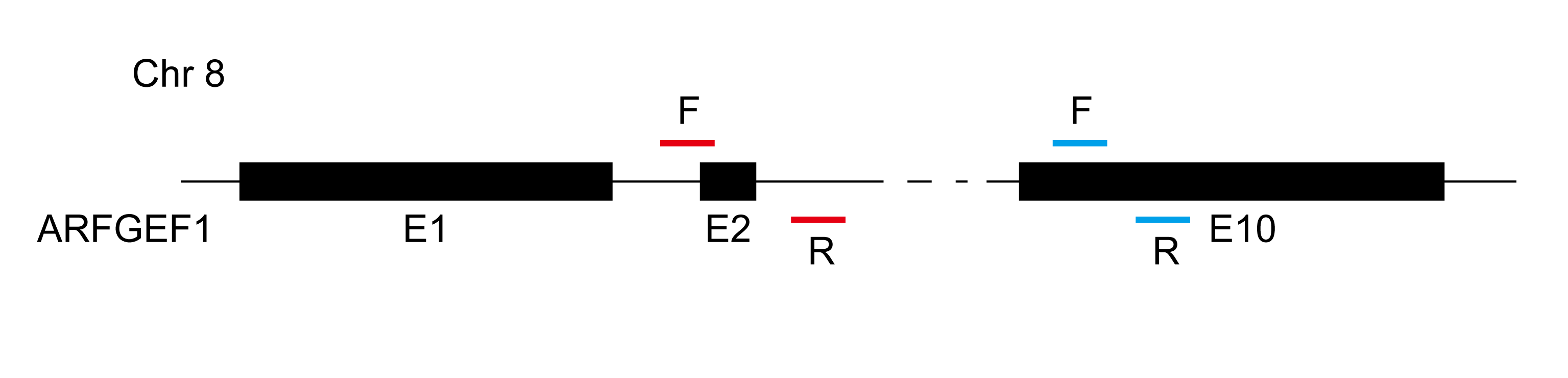

Supplement: S2 Fig — The red lines indicate the locations of forward and reverse primers for pre-mRNA of ARFGEF1, and the blues ones indicate the locations of primers for mature mRNA of ARFGEF1. (TIF) [file ppat.1009294.s002.tif]

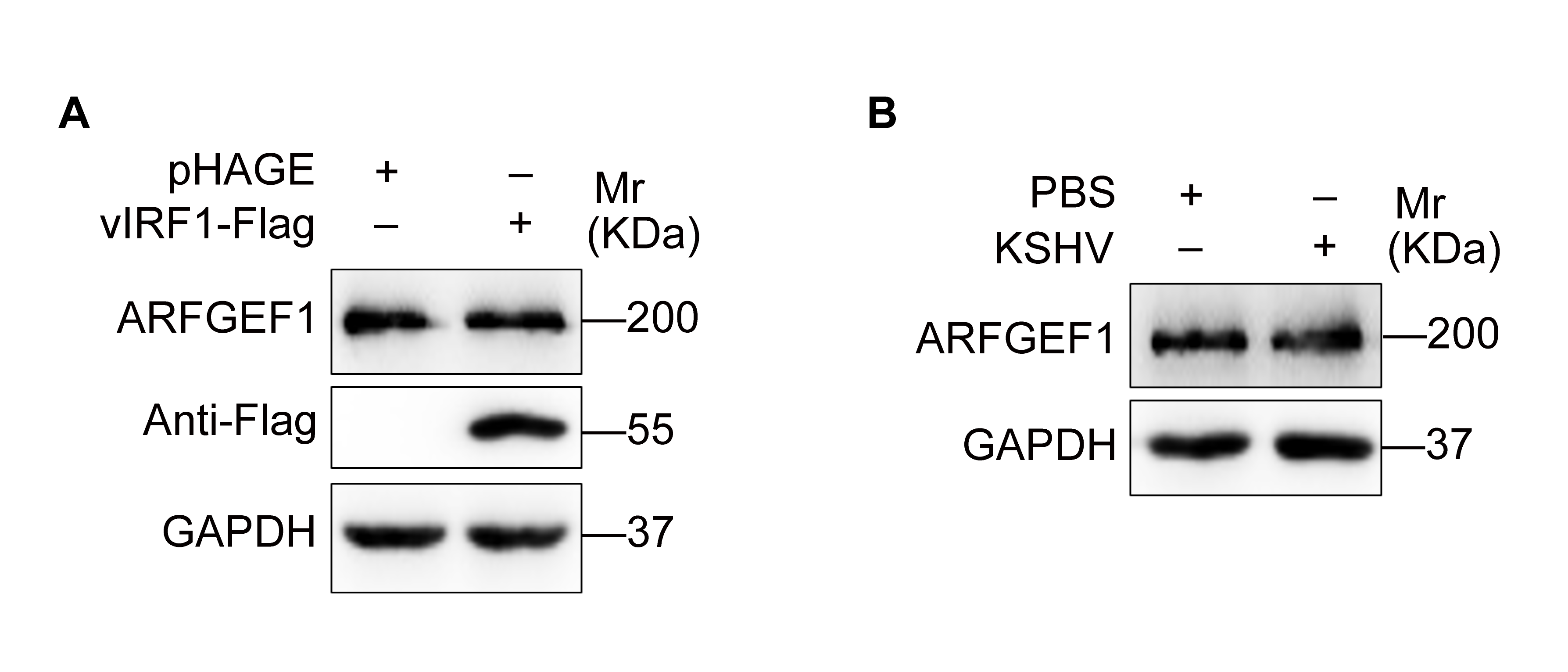

Supplement: S3 Fig — (A). ARFGEF1 was detected after ectopic expression of vIRF1 in EA.hy926 cells via Western blotting. (B). ARFGEF1 was examined after KSHV (3 MOI) infection in EA.hy926 cells via Western blotting. (TIF) [file ppat.1009294.s003.tif]

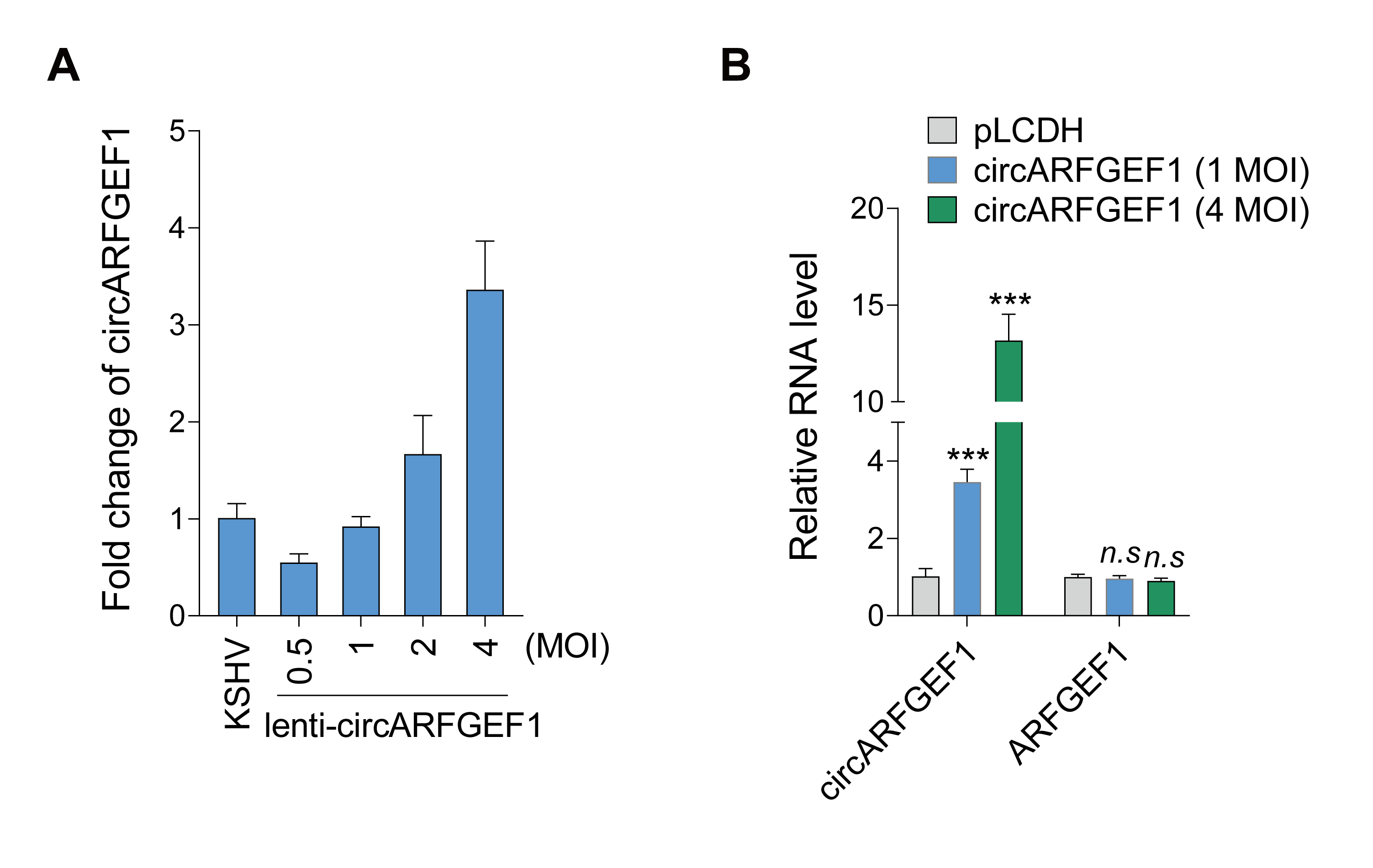

Supplement: S4 Fig — (A). qPCR results showing circARFGEF1 expression in EA.hy926 cells infected with KSHV or transduced with different MOI of lentiviral circARFGEF1. The level of circARFGEF1 in KSHV cells was set as “1” for comparison. (B). qPCR results of circARFGEF1 and mRNA of its parental gene ARFGEF1 in EA.hy926 cells transduced with lentiviral circARFGEF1 at 1 or 4 MOI and its control pLCDH. Data were shown as mean ± SD. *** P < 0.001, Student’s t-test. n.s, not significant. (TIF) [file ppat.1009294.s004.tif]

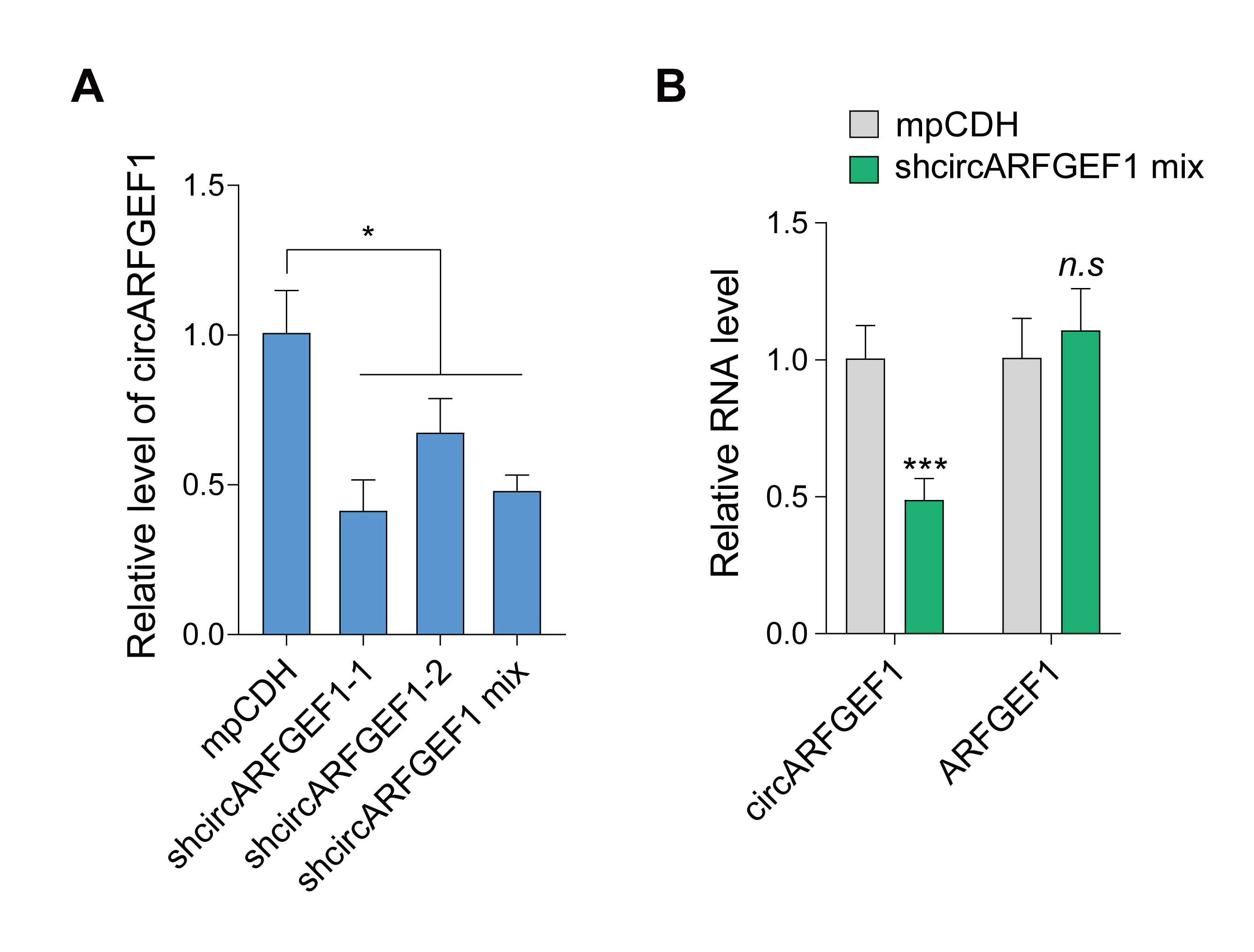

Supplement: S5 Fig — (A). circARFGEF1 was silenced by lentivirus-mediated two shRNAs targeting circARFGEF1 and the knockdown efficiency of circARFGEF1 was measured by qPCR. (B). circARFGEF1 and mRNA level of its parental gene ARFGEF1 in EA.hy926 cells transduced with lentivirus-mediated shRNAs mix targeting circARFGEF1 or the control mpCDH. Data were shown as mean ± SD. * P < 0.05; *** P < 0.001, Student’s t-test. n.s, not significant. (TIF) [file ppat.1009294.s005.tif]

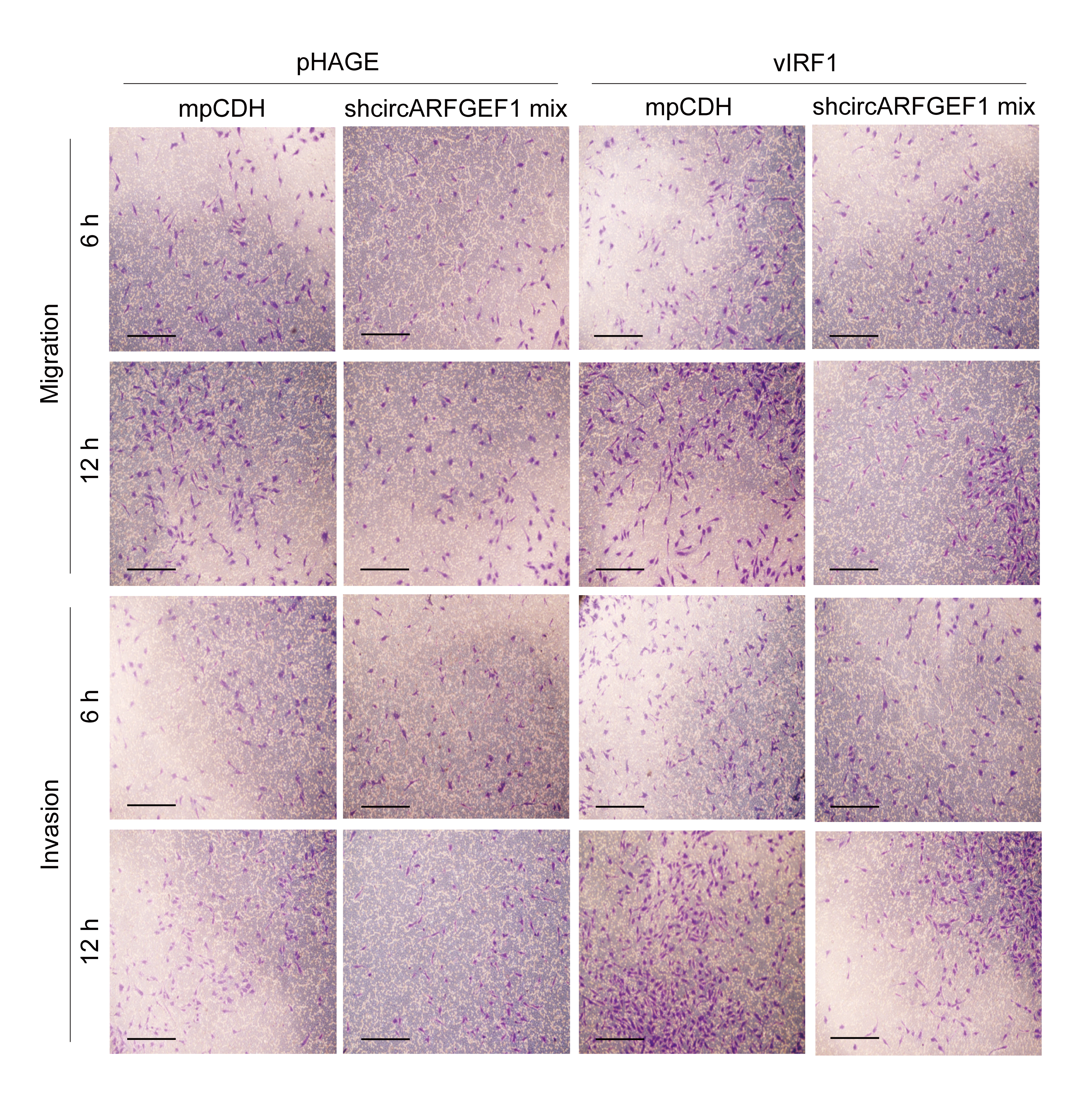

Supplement: S6 Fig — Lentiviral vIRF1 transduced pri-HUVECs were further transduced with a mixture of shRNAs targeting circARFGEF1 (shcircARFGEF1 mix), and were subjected to Transwell migration and invasion assays described in the “Materials and methods” section. The migrated and invaded cells were counted at 6 h and 12 h post seeding. Representational photographs of migration and invasion were exhibited (original magnification, ×100). Quantification of Transwell migration and invasion assay was described in Fig 3B and 3C. (TIF) [file ppat.1009294.s006.tif]

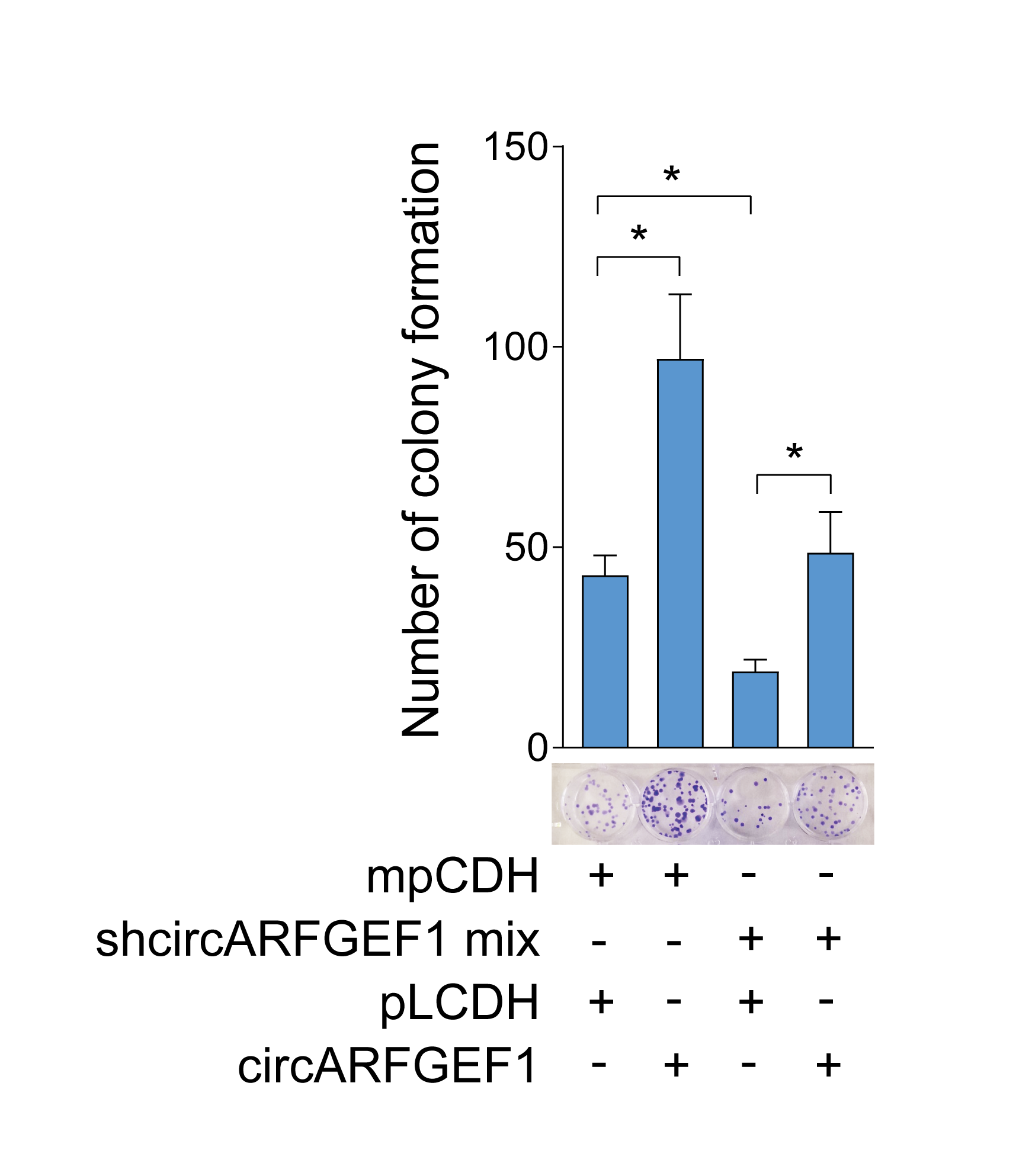

Supplement: S7 Fig — EA.hy926 cells were transduced with lentivirus-mediated shcircARFGEF1 mix targeting circARFGEF1, and further transduced with lentivirus-mediated overexpression of circARFGEF1. Then cells were subjected to cell plate colony formation, which was fully described in “Materials and Methods” section. * P < 0.05, Statistical significance was determined using one-way ANOVA followed by Tukey’s multiple comparisons test. (TIF) [file ppat.1009294.s007.tif]

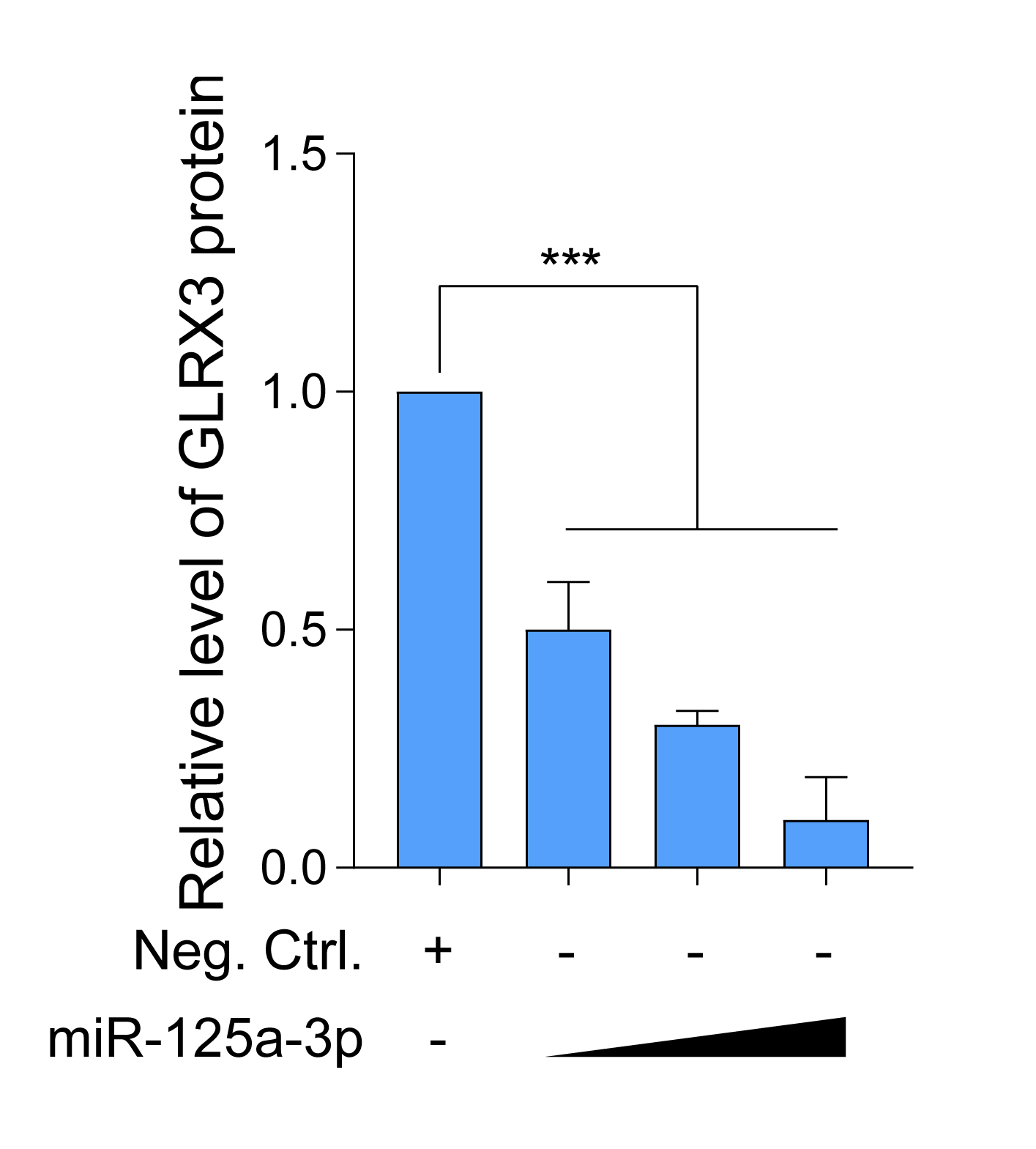

Supplement: S8 Fig — GLRX3 protein expression in EA.hy926 cells transfected with increasing amounts of miR-125a-3p mimic (10, 20 and 50 nM) or its control (Neg. Ctrl.) for 48 h was quantified in Fig 6F. The difference of GLRX3 reduction was analyzed for three independent experiments. *** P < 0.001, Student’s t-test. (TIF) [file ppat.1009294.s008.tif]

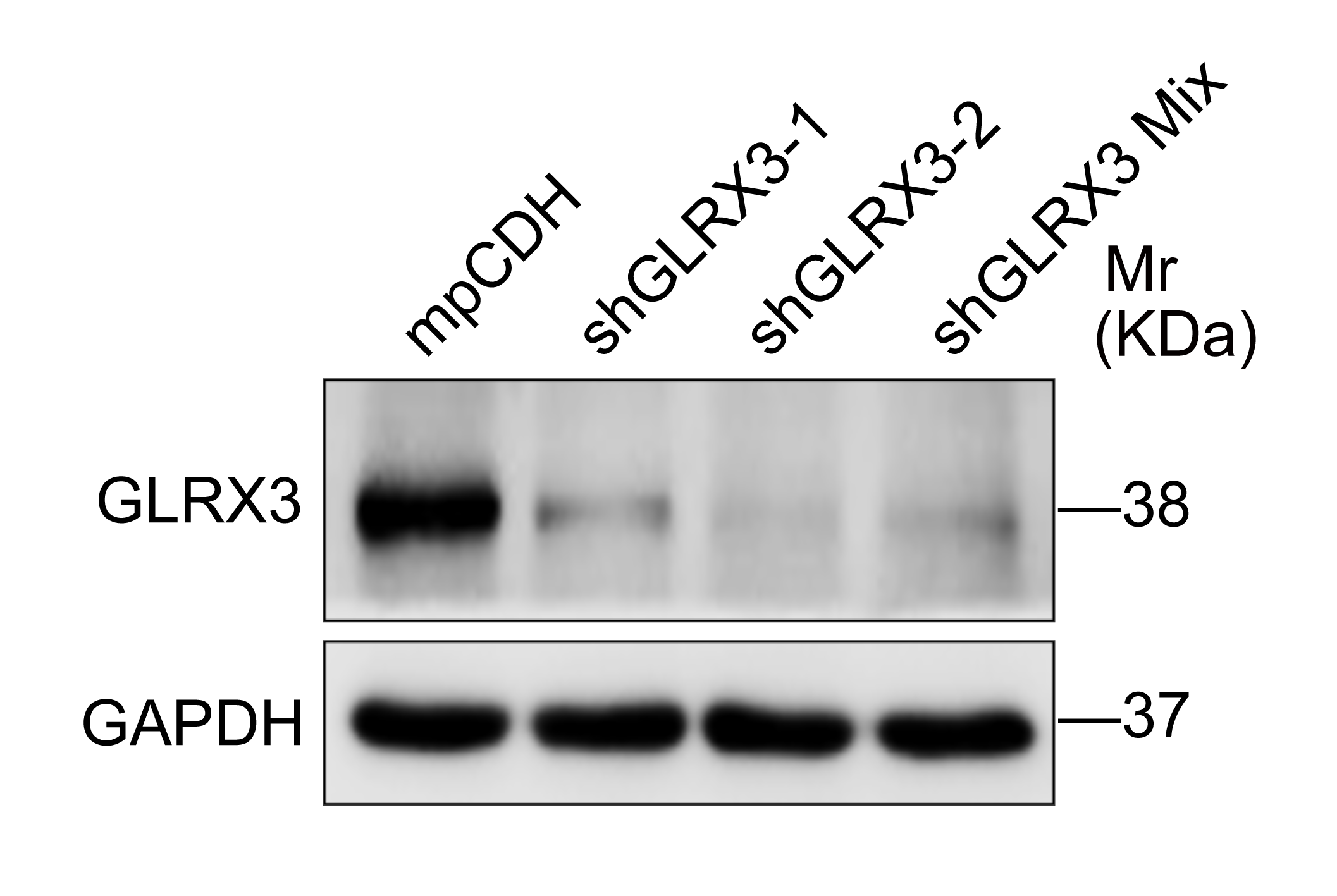

Supplement: S9 Fig — Western blotting was performed with the indicated antibodies in EA.hy926 cells transduced with lentiviruses containing shRNA 1 and 2, and a mixture of the two shRNAs targeting GLRX3 or the control mpCDH. Experiments were independently repeated three times with similar results. Results shown were from a representative experiment. (TIF) [file ppat.1009294.s009.tif]

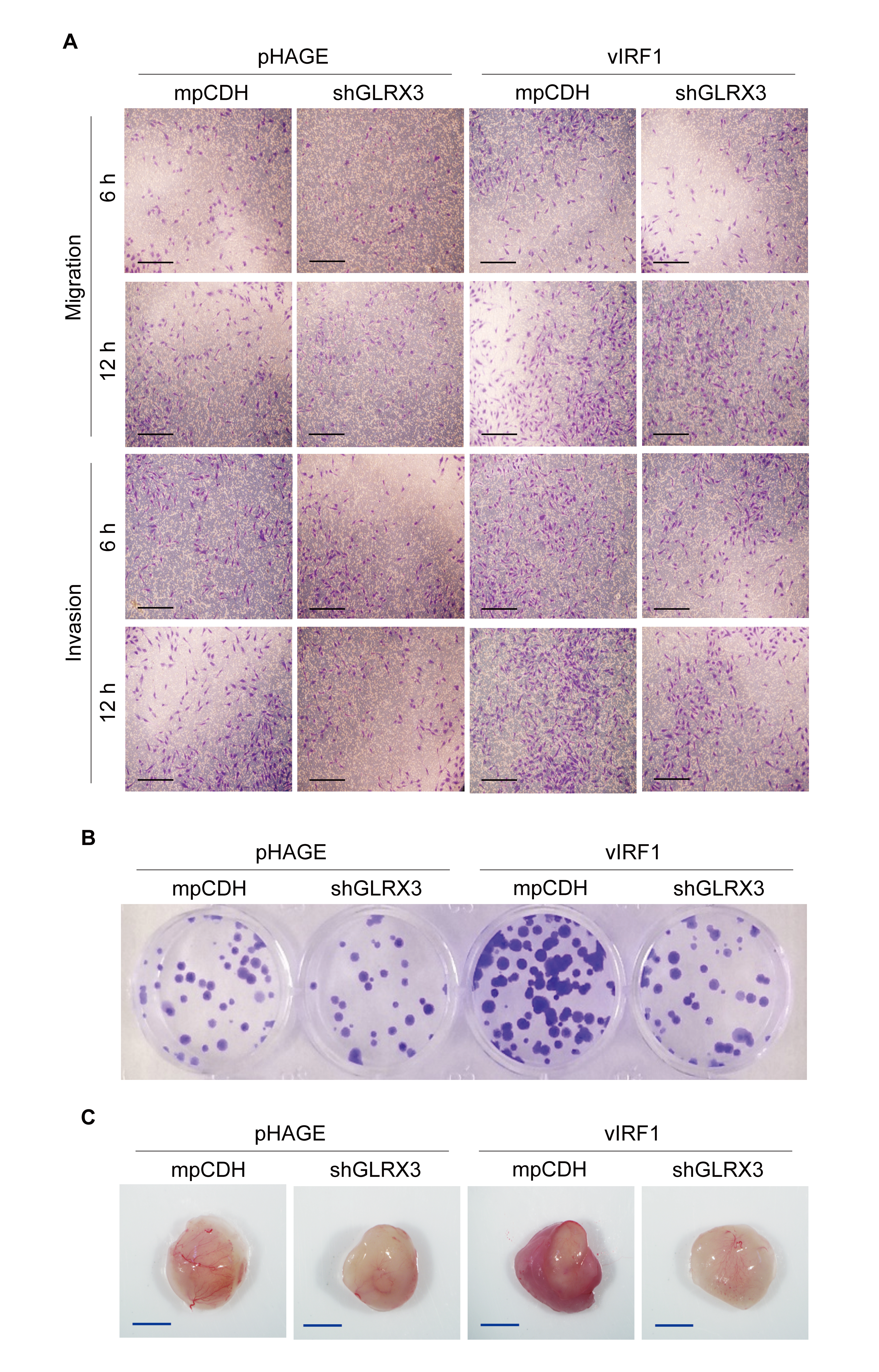

Supplement: S10 Fig — (A). GLRX3 was interfered by two different shRNAs in vIRF1 transduced pri-HUVECs. Cells were subjected to Transwell migration and invasion assay described in the “Materials and methods” section. The migrated and invaded cells were counted at 6 h and 12 h post seeding. Representational photographs of migration and invasion were exhibited (original magnification, ×100). Quantification of Transwell migration and invasion assay was described in Fig 7H and 7I. (B). Plate colony formation assay of EA.hy926 cells treated as in (A) was performed as described in the “Materials and methods” section. Representational photographs of plate colony were exhibited. Quantification of plate colony formation assay was described in Fig 7J. (C). The mixture containing high concentration Matrigel and EA.hy926 cells treated as in (A) was injected into nude mice. The details were shown in the “Materials and methods” section. Representational photographs of plugs were exhibited. Scar bars, 1 cm. Quantification of hemoglobin in plug tissues was described in Fig 7K. (TIF) [file ppat.1009294.s010.tif]

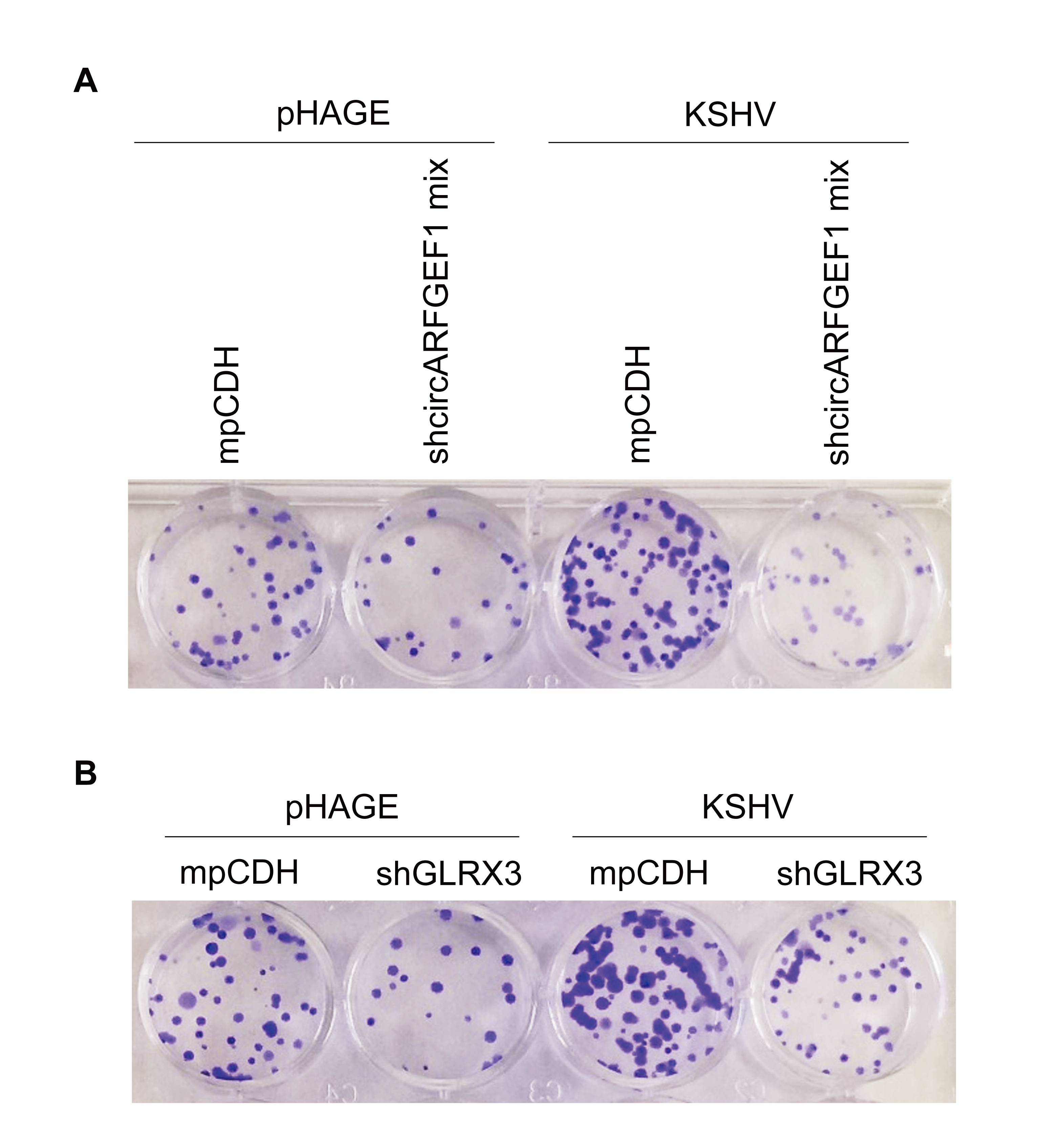

Supplement: S11 Fig — (A). Plate colony formation analysis of EA.hy926 cells treated with PBS (PBS), infected with KSHV wild type virus (3 MOI) or transduced with lentivirus-mediated shcircARFGEF1 sequences targeting circARFGEF1. Plate colony formation assay was performed as described in the “Materials and methods” section. Quantification of plate colony formation assay was described in Fig 8D. (B). Plate colony formation analysis of EA.hy926 cells treated with PBS (PBS), infected with KSHV wild type virus (3 MOI) or transduced with lentivirus-mediated shGLRX3 targeting GLRX3. Plate colony formation assay was performed as described in the “Materials and methods” section. Quantification of plate colony formation assay was described in Fig 8G. (TIF) [file ppat.1009294.s011.tif]
