# Supplementary material for: CircRNA ARFGEF1 functions as a ceRNA to promote oncogenic KSHV-encoded viral interferon regulatory factor induction of cell invasion and angiogenesis by upregulating glutaredoxin 3
Source: PLoS Pathog. 2021 Feb 4;17(2):e1009294. doi: 10.1371/journal.ppat.1009294 (PMC7888650; doi:10.1371/journal.ppat.1009294)
Supplement: S2 Table — (DOCX) [file ppat.1009294.s013.docx]

**S2 Table.** The sequences of the shRNAs

| Target | Application | Sequence (5’ to 3’) |
| --- | --- | --- |
| circARFGEF1  GLRX3  Lef1 | shRNA #1  shRNA #2  shRNA #1  shRNA #2  shRNA #3  shRNA #1  shRNA #2 | GATAATAAAGAGGAAATAAA  ATAAAGAGGAAATAAAAGC  CGAAGTTATGGCAGAGTTA  GAACCACGCTGTGGTTTCA  GCTTACTCAAATTGGCCAA  ATAAAGCTGCATATGTAGCTG  GGCTAAATATTATGAATTAGC |
