# Supplementary material for: CircRNA ARFGEF1 functions as a ceRNA to promote oncogenic KSHV-encoded viral interferon regulatory factor induction of cell invasion and angiogenesis by upregulating glutaredoxin 3
Source: PLoS Pathog. 2021 Feb 4;17(2):e1009294. doi: 10.1371/journal.ppat.1009294 (PMC7888650; doi:10.1371/journal.ppat.1009294)
Supplement: S3 Table — (DOCX) [file ppat.1009294.s014.docx]

**S3 Table.** The sequences of specific primers of RT-qPCR.

| Target | Application | Sequence (5’ to 3’) |
| --- | --- | --- |
| circARFGEF1  GLRX3  GAPDH  ARFGEF1  pre-ARFGEF1  β-Actin  U6  circARFGEF1 | RT-qPCR  RT-qPCR  RT-qPCR  RT-qPCR  RT-qPCR  RT-qPCR  RT-qPCR  ChIP-qPCR | F: CTTATTGCTTATGGGCACT  R: AGTTTCCGCTTTTATTTCCT  F: GGGACTCAAAGCCTATTCCAG  R: TTTGTCAGCACTTTGAGCCTT  F: GAAGGTGAAGGTCGGAGTC  R: GAAGATGGTGATGGGATTTCC  F: GCAGTATCTTTGTGTTGCACTC  R: CATCTGCACAAATCCTCGTCA  F: CCCCTCAGAGGAAATAAAAGC  R: CTCACATGCAGAGAATTTGCC  F: TTGCCGACAGGATGCAGAAGGA  R: AGGTGGACAGCGAGGCCAGGAT  F: CTCGCTTCGGCAGCACA  R: AACGCTTCACGAATTTGCGT  F: GGTTTACATGTCCTGGAC  R: CAACTCTAGATTTCAGGTCC |
